# Differential microbial community assembly following co-housing versus microbiota transplant

**DOI:** 10.1093/ismejo/wraf256

**Published:** 2025-11-17

**Authors:** James S Weagley, Luis Alberto Chica Cárdenas, Ana Romani, Meagan E Sullender, Somya Aggarwal, Heyde Makimaa, Michael P Hogarty, Rachel Rodgers, Elizabeth A Kennedy, Lynne Foster, Lawrence A Schriefer, Megan T Baldridge

**Affiliations:** Department of Medicine, Division of Infectious Diseases, Edison Family Center for Genome Sciences & Systems Biology, Washington University School of Medicine, St. Louis, MO 63110, United States; Department of Medicine, Division of Infectious Diseases, Edison Family Center for Genome Sciences & Systems Biology, Washington University School of Medicine, St. Louis, MO 63110, United States; Department of Medicine, Division of Infectious Diseases, Edison Family Center for Genome Sciences & Systems Biology, Washington University School of Medicine, St. Louis, MO 63110, United States; Department of Medicine, Division of Infectious Diseases, Edison Family Center for Genome Sciences & Systems Biology, Washington University School of Medicine, St. Louis, MO 63110, United States; Department of Medicine, Division of Infectious Diseases, Edison Family Center for Genome Sciences & Systems Biology, Washington University School of Medicine, St. Louis, MO 63110, United States; Department of Medicine, Division of Infectious Diseases, Edison Family Center for Genome Sciences & Systems Biology, Washington University School of Medicine, St. Louis, MO 63110, United States; Department of Medicine, Division of Infectious Diseases, Edison Family Center for Genome Sciences & Systems Biology, Washington University School of Medicine, St. Louis, MO 63110, United States; Department of Medicine, Division of Infectious Diseases, Edison Family Center for Genome Sciences & Systems Biology, Washington University School of Medicine, St. Louis, MO 63110, United States; Department of Pediatrics, Washington University School of Medicine, St. Louis, MO 63110, United States; Department of Medicine, Division of Infectious Diseases, Edison Family Center for Genome Sciences & Systems Biology, Washington University School of Medicine, St. Louis, MO 63110, United States; Department of Medicine, Division of Infectious Diseases, Edison Family Center for Genome Sciences & Systems Biology, Washington University School of Medicine, St. Louis, MO 63110, United States; Department of Medicine, Division of Infectious Diseases, Edison Family Center for Genome Sciences & Systems Biology, Washington University School of Medicine, St. Louis, MO 63110, United States; Department of Medicine, Division of Infectious Diseases, Edison Family Center for Genome Sciences & Systems Biology, Washington University School of Medicine, St. Louis, MO 63110, United States; Department of Molecular Microbiology, Washington University School of Medicine, St. Louis, MO 63110, United States

**Keywords:** microbiota transplantation, microbial community assembly, virome dynamics, murine models, gut microbial ecology, antibiotic perturbation and recovery, spatial and temporal microbial dynamics

## Abstract

Mouse models are vital tools for discerning the relative contributions of host and microbial genetics to disease, often requiring the transfer of microbiota between different mouse strains. Transfer methods include antibiotic treatment of recipients and colonization using either co-housing with donors or the transplantation of faecal or caecal donor material. However, the efficiency and dynamics of these methods in reconstituting recipients with donor microbes is not well understood. We thus directly compared co-housing, faecal transplantation, and caecal transplantation methods. Donor mice from Taconic Biosciences, possessing distinct microbial communities, served as the microbial source for recipient mice from Jackson Laboratories, which were treated with antibiotics to disrupt their native microbiota. We monitored bacterial and viral populations longitudinally over the course of antibiotics treatment and reconstitution using 16S rRNA gene sequencing, quantitative PCR (qPCR), and shotgun sequencing of viral-like particles (VLPs). As expected, antibiotic treatment rapidly depleted microbial biomass and diversity, with slow and incomplete natural recovery of the microbiota in non-transfer-recipient control mice. Although all transfer methods reconstituted recipient mice with donor microbiota, co-housing achieved this more rapidly for both bacterial and viral communities. Overall, faecal and caecal transplant resulted in highly similar colonization processes with some minor variation in enrichment for two specific bacterial families. This study provides valuable insights into microbial ecology, as well as the dynamics underlying experimental microbial transfer methods, enhancing reproducibility and informing best practices for microbiota transfer in mouse models.

## Introduction

As appreciation grows for the diverse range of phenotypes the microbiota can mediate so does our reliance on manipulatable small animal models to experimentally interrogate these effects. The gut microbiota, a complex community of microorganisms residing in the gastrointestinal tract, plays a pivotal role in host health and physiology [1, 2]. These microbial communities are involved in critical functions such as nutrient metabolism [3, 4], immune system development [5–7], and protection against pathogens [8–10]. Dysbiosis, or the disruption of the normal microbiota balance, has been implicated in a range of diseases including obesity [11, 12], malnutrition [13, 14], and inflammatory bowel diseases [15, 16]. Understanding the intricate relationships between the microbiota and host systems is therefore essential for developing therapeutic strategies targeting microbiota-related diseases.

To unravel these complex host–microbe interactions, researchers heavily leverage animal models, particularly mice. Controlled studies using mouse models enable the manipulation of microbial communities to observe resultant effects on host physiology and disease outcomes. A key aspect of such research is the generation of gnotobiotic mice—animals that are germ-free or colonized with specific, defined microbial communities [17]. These models provide a unique platform to study the microbiota’s role under controlled conditions. Despite the utility of germ-free mice for numerous applications, however, their use is associated with some drawbacks. For investigators studying numerous mouse genotypes, executing experiments not amenable to the use of a gnotobiotic isolator such as those involving particular pathogens or behavioural testing, or performing studies requiring a normal microbiota during development which is then disrupted, germ-free mice may be impractical or inappropriate. For instance, exposure, or lack thereof, to specific microbes during development results in altered immune and metabolic status in mice that may impact numerous phenotypes of interest [5–7, 18–20]. Antibiotic treatment represents a cost-effective and accessible alternative for the depletion of the microbiota, and this approach has been broadly used by many investigators to examine many of the same phenotypes explored in germ-free mice, often with strong concordance between the two models [17, 21, 22]. Not only is this an important experimental method, but antibiotic treatment provides a relevant model of large-scale ecological disturbances in microbial ecosystems.

Establishing gnotobiotic mice or reconstituting the microbiota after perturbation necessitates reliable and efficient methods for microbial transfer. Common techniques include faecal microbiota transplantation, caecal content transplantation, and co-housing. Faecal transplantation involves transferring faecal material from a donor to a recipient via the oral route, whereas caecal transplantation is similar but utilizes the caecal contents, which may have a different microbiota. Co-housing leverages the natural coprophagic behaviour of mice to transfer microbes. Co-housing also models the natural horizontal transmission of microbes through social contact. Social transmission of microbes has been described not only in coprophagic species such as mice, but also in humans and other animals, where shared environments and social interactions shape microbial communities [23–26]. Although the direct mechanisms of transmission vary across host species, co-housing provides an ecologically relevant model for how microbes are spread in these social settings [27, 28]. Despite their widespread use, experiments designed utilizing these methods are generally based on historical precedent rather than a comprehensive understanding of their efficacy or the specific dynamics of microbial colonization they promote. Further, comparing these transfer methods provides unique insights into the dynamics of post-disturbance ecosystem recovery with and without intervention [26, 29].

This reliance on convention can lead to assumptions that impact experimental outcomes and reproducibility, a major challenge facing the field [30–33]. For instance, variation in microbial transfer efficiency can affect the establishment of the desired microbiota, influencing the host’s physiological responses and potentially skewing results [34–36]. Furthermore, although faecal bacterial community dynamics have been studied following faecal transplant and co-housing, the transfer and establishment of viral communities (the virome) remain less understood [37]. Given the growing recognition that bacteriophages and other viruses shape microbial communities and influence host health [38–44], and that phages can also move across host social networks [45], it is crucial to consider both bacterial and viral components when evaluating these methods. Additionally, variation in microbial community composition across gastrointestinal sites following these various transfer methods has not been well characterized.

Herein, we systematically compare faecal transplantation, caecal transplantation, and co-housing as methods for transferring microbes to microbially depleted mice, characterizing this process longitudinally. Recipient mice from Jackson Laboratories were treated with antibiotics for one week to disrupt their native microbiota. Donor mice from Taconic Biosciences, known to have distinct microbial communities from Jackson, served as the source of bacteria and viruses for transfer [46]. By employing 16S rRNA gene sequencing and quantitative PCR (qPCR), we monitored bacterial load, diversity, and composition. Additionally, we analyzed viral community composition and diversity through short-read shotgun sequencing of viral-like particles (VLPs). We employed a comprehensive contig-based, annotation-agnostic strategy in conjunction with in-depth classification and binning of VLP sequences, allowing for an unbiased characterization of both known and novel viruses. Thus, we provide a more complete landscape of virome dynamics than would be possible with annotation-dependent methods alone.

We find that co-housing facilitates a more rapid transfer of both bacterial and viral communities compared to faecal and caecal transplants, which performed similarly to each other. Viral communities generally tracked with bacterial colonization, though each method enriched for distinct bacterial taxa—and their associated phages—at different timepoints. These results highlight method-specific influences on microbial community dynamics and underscore the importance of method selection in experimental design.

By carefully characterizing these commonly used methods, our study provides valuable insights that can enhance reproducibility and consistency in microbiota research. This work informs best practices for replacing the enteric microbiota of mice and contributes to our understanding of the mechanisms underlying microbial transfer and establishment in experimental models. Such insights are essential for advancing microbiota research and for the development of interventions targeting microbiota-related diseases.

## Materials and methods

### Mice and treatments

WT C57BL/6 J (stock no. 000664) mice were purchased from Jackson Laboratories (JAX) and C57BL/6 N (stock #B6), murine pathogen free health standard, mice were purchased from Taconic Biosciences (TAC). These mice were maintained at Washington University School of Medicine under specific-pathogen-free conditions according to University guidelines. Mice were acclimated for 5-20 days on campus following delivery from vendors. Female mice, aged six to eight weeks, were exclusively used in all experiments to facilitate co-housing experiments. Experimental mice in all groups were housed with up to two mice of the same sex per cage with autoclaved standard chow pellets and water provided *ad libitum*. Cages of female mice were treated at 6 weeks of age with an antibiotic cocktail [1 g/l ampicillin, 1 g/l neomycin, 0.5 g/l vancomycin (Sigma, St. Louis, MO)] in drinking water for 7 days. Donor, recipient, and control mice were age-matched.

For faecal and caecal transplants, donor mice were euthanized by CO₂ asphyxiation, and dissections were performed under sterile conditions in a biosafety cabinet using Clidox- and ethanol-sterilized tools. Faecal pellets or caecal contents were collected into sterile 50 ml conical tubes and homogenized in phosphate-buffered saline. Resulting homogenates were administered to recipient mice by oral gavage using sterile syringes and 20-gauge curved gavage needles on day 8 and day 9, with each mouse receiving ~200–400 μL. Faecal samples were collected prior to gavage on day 9. For co-housing, Jackson recipient mice were paired with Taconic donor mice on Day 8. Co-housed pairs were maintained in fresh cages with autoclaved bedding, food, and water.

Each experimental replicate included control groups: untreated JAX and TAC mice and antibiotic-treated JAX mice with no subsequent experimental transfer. Faecal samples were collected longitudinally, and all mice were monitored daily for behaviour, health status, and cage conditions. Special care was taken to prevent cage flooding and ensure adequate chow and water access, particularly during antibiotic treatment. After transplantation or co-housing, all mice received autoclaved drinking water without antibiotics for the remainder of the experiment. All animal procedures were approved by and conducted in compliance with Washington University’s Institutional Animal Care and Use Committee guidelines. The experiment was repeated for a total of three independent experiments, with data combined in subsequent analyses.

### 16S rRNA gene sequencing and qPCR quantification of bacterial load

Phenol:chloroform-extracted DNA from faecal pellets was used for both 16S rRNA gene qPCR and sequencing. SYBR green qPCR for the 16S rRNA gene was performed with 515F (5′-GTGCCAGCMGCCGCGGTAA-3′) and 805R (5′-GACTACCAGGGTATCTAATCC-3′) primers to detect the V4 hypervariable region. Absolute copies were quantified based on a standard curve.

For short read sequencing, samples were prepared in three batches with samples from across experiments and treatment groups semi-randomized, in a supervised manner, between the pools. Primer selection and PCRs were performed as described previously [14]. Briefly, each sample was amplified in triplicate with Golay-barcoded primers specific for the V4 region (F515/R806), combined, and confirmed by gel electrophoresis. Negative controls were included for DNA extraction and 16S rRNA gene amplicon library preparation; these did not show bands following PCR amplification and were not included in sequencing pools. PCR amplifications contained 18.8 μL RNase/DNase-free water, 2.5 μL 10X High Fidelity PCR Buffer (Invitrogen), 0.5 μL 10 mM dNTPs, 1 μL 50 mM MgSO4, 0.5 μL each of the forward and reverse primers (10 μM final concentration), 0.1 μL Platinum High Fidelity Taq (Invitrogen) and 1.0 μL genomic DNA. Reactions were held at 94°C for 2 min to denature the DNA, with amplification proceeding for 26 cycles at 94°C for 15 s, 50°C for 30 s, and 68°C for 30 s; a final extension of 2 min at 68°C was added to ensure complete amplification. Amplicons were pooled and purified with 0.6× Agencourt Ampure XP beads (Beckman-Coulter) according to the manufacturer’s instructions. The final pooled samples, along with aliquots of the sequencing primers, were sent to the Center for Genome Sciences (Washington University School of Medicine) for sequencing by the 2×250 bp protocol with the MiSeq System (Illumina).

The resulting FASTQ files were processed using the DADA2 pipeline (v1.28.0) implemented in Nephele (version 2024_Jan_16, commit: 71b6a72) [47]. Initial quality assessment was conducted to determine appropriate truncation and filtering parameters. Reads were trimmed to remove low-quality bases and sequencing primers and truncated based on quality scores to ensure high-confidence base calls [48, 49]. Specifically, reads were truncated at the position where the quality score dropped below a threshold (default: 4) [50, 51]. Reads with expected errors exceeding a set threshold (default: 5) were discarded to further enhance data quality. Forward and reverse reads were merged, requiring a minimum overlap of 12 bp and allowing for zero mismatches in the overlapping region to ensure accurate reconstruction of the target amplicon [52, 53]. Chimeric sequences were identified and removed using the consensus method to prevent false-positive operational taxonomic units.

Taxonomic classification was performed using the Ribosomal Database Project classifier, with a minimum bootstrap confidence threshold of 40 for taxonomic assignments and the SILVA v138.1 database [54]. Additionally, a phylogenetic tree was constructed using MAFFT for multiple sequence alignment and FastTree2 for tree building, facilitating downstream phylogenetic analyses [55, 56]. The output included an amplicon sequence variant (ASV) table, taxonomic assignments, and phylogenetic relationships, providing a comprehensive dataset for subsequent diversity and composition analyses. All analyses were conducted using default parameters unless otherwise specified, adhering to best practices for 16S rRNA gene sequence processing to ensure accurate and reproducible results. One sample (“TACNoT.MTP2.D11.74”) was excluded due to lack of stool at the time of DNA extraction – it was noted that there was no stool in this sample tube however it was processed alongside all other samples, and the data were excluded from downstream analyses. 12 ASVs assigned to Eukaryota, Mitochondria, or Chloroplast were removed, and one sample that no longer contained bacterial reads were excluded from downstream analysis.

### Bacterial ecological and distance analyses

Bacterial community composition and ecological metrics were assessed using both alpha and beta diversity analyses. Observed richness was calculated to evaluate within-sample diversity. For richness metrics, ASVs were grouped into bacterial species based on >97% sequence identity [47, 57]. Per-sample read count (sequencing depth) was not included as a covariate in α-diversity models. In our study, antibiotic treatment produced a marked reduction in bacterial biomass, which directly reduced recoverable reads; sequencing depth is therefore directly influenced by biological experimental variables, not an independent technical artifact. Adjusting for it would control away part of the treatment effect and bias between-group comparisons. To limit technical variability, samples were randomized across libraries/runs and processed with identical laboratory and bioinformatic workflows under uniform quality thresholds. α-diversity was calculated on the resulting feature tables and between-group tests were performed without a depth covariate. Where metrics are known to be depth-sensitive (e.g. richness-type measures), we interpret them in light of the treatment-induced depth shift and present results transparently under the same processing settings for all groups.

Beta diversity was assessed using unweighted UniFrac distances to capture phylogenetic differences between samples. A phylogenetically informed β-diversity metric was used as many ecologically relevant microbial traits exhibit measurable phylogenetic signal; accounting for shared ancestry provides a functionally meaningful summary of community change (i.e. replacements among close relatives, which likely share niches, are treated as smaller shifts than replacements among distant clades). Our aim was not to necessarily to infer within-host evolution or macroscale biogeography, but to obtain an ecologically interpretable measure in which ancestry approximates trait similarity relevant to processes such as niche overlap/competition. These analyses were implemented using the “phyloseq” and “vegan” R packages, which offer comprehensive tools for ecological and diversity analyses. Principal coordinate analysis (PCoA) was performed to visualize patterns in beta diversity across treatment groups and time points [58, 59]. Pairwise comparisons between groups were conducted using PERMANOVA with Bonferroni corrections to identify statistically significant differences (alpha = 0.05). These analyses provided insights into the ecological shifts in bacterial communities associated with different transfer methods and recovery stages. All figures were generated in R using ggplot2 [60].

### Generation of compositional data

To generate compositional (relative abundance) data, counts from individual ASVs or viral contigs were aggregated using the “tax_glom” function in phyloseq. Aggregation occurred at the family level for bacterial ASV data, and by predicted source for viral contigs. The “transform_sample_counts” function was used to divide the individual aggregated counts by the total read count for the samples to obtain relative abundance for all taxa in a given sample.

### Identifying taxa associated with each vendor

Bacterial taxa specific to each vendor were identified using ASVs derived from 16S rRNA gene sequencing. Taxa were classified as Jackson-associated or Taconic-associated based on their differential prevalence and abundance in faecal samples collected from untreated mice during the first week of the experiment (days 0–7). Samples were limited to those without antibiotic treatment to accurately capture baseline community differences. Differential abundance analyses were conducted using the DESeq2 pipeline. Significant differences were determined using an adjusted *P* value threshold of .05. Prevalence was calculated as the proportion of samples in which a given ASV was detected within each vendor group.

To incorporate abundance and prevalence, a combined fold-change metric was calculated by summing the log2(fold change) from differential abundance analysis in DESeq2 and the log2(prevalence ratio) between the two groups, with combined score cutoff of ±4 and a DESeq2 *P* < .05. ASVs that did not meet these criteria were further subclassified as Neither (early/present) if they were detected in untreated controls but not significantly associated with either source community, or Neither (absent) if they were not observed in any controls during the first week.

### Predicting functional capacity of bacterial communities

Functional capacities of bacterial communities were inferred from 16S rRNA gene sequencing data using the PICRUSt2 (v. 2.5.3) pipeline. ASVs were mapped to predicted genes and metabolic pathways using the MetaCyc database. Differentially abundant pathways were identified using DESeq2 with an alpha threshold of 0.0005. Comparisons were made between antibiotic-treated recipient mice (co-housed, faecal transplantation, or caecal transplantation groups) and control communities from untreated Jackson and Taconic mice. Functional convergence over time was assessed across treatment groups to evaluate the recovery of microbial metabolic potential.

### Taxonomic changes following microbial transfer

Changes in bacterial taxonomic composition were assessed longitudinally using 16S rRNA gene sequencing. ASVs were classified into taxonomic families, and relative abundances were tracked over time in recipient mice across different transfer methods (co-housing, faecal, and caecal transplant). Differentially enriched taxa were identified using ANCOM-BC2 (v. 2.6.0) [61]. Taxonomic dynamics were visualized using stacked bar plots.

### Statistical comparisons across gastrointestinal sites

To evaluate differences in observed species richness across experimental groups, we fit a linear mixed-effects model using the lmer function from the lme4 package (v. 1.1-35.5), with Sample Type (gastrointestinal site) and Condition (transfer method) specified as fixed effects and Sequencing Library included as a random effect to account for variation across sequencing batches [58, 62]. ANOVA as implemented in the lmerTest package (3.1-3) was used to test the significance of fixed effects [63]. Post hoc comparisons of estimated marginal means were conducted using the lsmeans function within the emmeans package (v. 1.10.3) with Tukey’s HSD correction for multiple testing [64]. Group-level differences were visualized using compact letter display from the multcompView package (v. 0.1-10), where groups not sharing a letter are significantly different at α = 0.05 [65].

### VLP isolation, gDNA extraction, virome sequencing and analysis

For VLP isolation, stool pellets were homogenized in SM buffer and centrifuged at 9000 × g for 10 min. The resulting supernatant was collected and sequentially passed through 0.45 μm PES and 0.22 μm filters. The filtrate was then treated with DNase and lysozyme to degrade bacterial DNA, RNA, and other contaminants, after which the reaction was terminated by the addition of EDTA. Viral DNA was subsequently extracted using the SDS/CTAB method, followed by Ampure bead clean-up. Short read sequencing libraries were prepared using standard Nextera protocols.

Sequencing was performed on a NextSeq System (Illumina), generating paired-end reads. Reads processed using the Hecatomb pipeline (Dec2021) and assembled into contigs using Flye (v. 2.9.1), which is optimized for accurate assembly of viral genomes from metagenomic data [66, 67]. Viral contigs were identified using stringent criteria for viral hallmark genes and annotated taxonomically using Cenote-Taker2 (v. 2.1.1), a tool designed for comprehensive identification and annotation of eukaryotic and prokaryotic viruses [68].

To account for batch effects associated with preparation of viral sequencing pools, raw viral count tables were first converted to a DESeq2 [69] dataset using the phyloseq_to_deseq2 [59] function with a design formula including VP, Source, Day, Antibiotics, and transfer status. Counts were log-transformed using variance-stabilizing transformation (VST) with blind = FALSE. Batch effects attributable to viral preparation were then removed using the removeBatchEffect function from the limma package, specifying the model design to preserve variation due to biological covariates [70]. The resulting batch-corrected abundances were rounded to the nearest integer, and any negative values were set to zero before being re-integrated into the phyloseq object for downstream analysis. Samples where fewer than 10 unique viral contigs were detected were filtered out of subsequent analyses.

### Viral ecological and distance analyses

Viral community composition and ecological dynamics were assessed using alpha (richness) and beta diversity measures (Jaccard distances). We were not able to generate phylogenetic distance estimates from our viral data, perhaps due to their predicted polyphyletic origins [71]. This precluded the use of phylogenetically informed distance metrics, like UniFrac, and we instead used Jaccard distance for viral beta diversity estimates. These analyses were implemented using the “phyloseq” and “vegan” R packages, which provide robust tools for microbial ecological analyses. PCoA was conducted to visualize temporal changes in viral community structure across treatment groups. Pairwise comparisons between groups were performed using PERMANOVA with Bonferroni corrections for multiple comparisons (alpha = 0.05). Viral communities were further classified as Jackson-associated, Taconic-associated, or non-supplier-specific based on their prevalence and abundance in control communities. Source discriminatory viral contigs were defined using the combined fold-change metric described for bacteria above.

### Hierarchical clustering of viral contigs

We employed a hierarchical clustering approach to categorize viral contigs into taxonomic units based on amino acid identity (AAI) and average nucleotide identity (ANI). Initial protein-coding genes were predicted from input contigs using Prodigal [72] (v2.6.3; in metagenomic mode). All-versus-all blastp alignments (BLAST+ [73], v2.7.1) were performed to compute AAI values, filtered to retain pairs with ≥20% AAI, ≥10% shared genes, and ≥ 8 homologous proteins. These filtered edges underwent Markov Clustering (MCL [74] v22.282) with an inflation parameter of 1.2 to define family-level clusters. Each family cluster was recursively processed using stricter thresholds (≥40% AAI, ≥20% shared genes, ≥16 homologs; MCL inflation = 2.0) to resolve genus-level clusters. Finally, genus clusters were subjected to nucleotide-level ANI analysis via BLASTn and checkV’s anicalc.py (checkV [75] v1.0.1), with viral operational taxonomic units delineated at ≥95% ANI and ≥ 85% coverage.

### Viral classification and binning

To further identify viral contigs, we then applied a two-tier filtering approach to these binned contigs. First, any contig shorter than 1500 bp was excluded. Contigs were then assessed with VirSorter2 (retaining those with a viral score ≥ 50) and DeepVirFinder (retaining those with a score ≥ 0.7 and *P* ≤ .05) [76, 77]. Contigs that did not meet these primary criteria were retained if they contained more viral genes than host genes and harbored at least one viral hallmark gene as identified by Cenote Taker2 [68]. This pipeline yielded 2262 putative viral contigs. Taxonomic assignments of these viral contigs were determined using vContact3, whereas iPHoP and PHATyp were employed for host and lifestyle (temperate vs. virulent) predictions, respectively [78, 79]. CheckV was used to assess genome quality and completeness [75]. Contigs were binned using CoCoNet, which discards sequences shorter than 2048 base pairs by deafult [80].

Bins were classified based on the aggregated predictions of the contigs they contained. Bins were labeled by their inferred source community if they contained any contigs from one of the sources—Taconic-associated or Jackson-associated—and labeled “Neither/Both” otherwise. No bins contained both Taconic-associated and Jackson-associated contigs. Next, lifestyle predictions (virulent vs. temperate) were consolidated at the bin level by checking whether any contigs in a bin were classified as temperate or virulent, and no contigs within the bin were predicted to be of the other lifestyle. No lifestyle classification was assigned to bins with a mixture of predictions at the contig level. Of the classified bins, 165 were predicted to be temperate whereas 138 were predicted to be virulent. Finally, host family assignments were integrated by examining the taxonomic predictions for each contig within a bin: bins containing contigs from a single family were assigned that family, bins spanning multiple families in a single phylum were labeled with the phylum followed by “_spp,” and bins spanning multiple phyla were labeled “multi.”

Viral sources were classifies as described above for bacterial ASVs. A combined fold-change metric incorporating abundance and prevalence, was calculated by summing the log2(fold change) from differential abundance analysis in DESeq2 and the log2(prevalence ratio) between the two groups, with combined score cutoff of ±4.5 and a DESeq2 *P* < .05.

### Selection of controls and limitations

Untreated TAC mice were not adequately sampled across gastrointestinal sites, or for virome sequencing (e.g. one sample post transfer). TAC co-housed mice have been used as a control for these analyses, recognizing that there is potential for JAX microbes to have transferred to these co-housed mice. Untreated TAC controls and TAC co-housed mice were not significantly different from each other in any analyses performed, at any timepoints where both were sampled. Additionally, there was no significant increase in the richness of JAX bacterial ASVs or viruses in TAC co-housed mice, providing support for this group as a reasonable control. We recognize this as a limitation of the study design and provide this explanation to maintain transparency.

### Use of large language models

Large language models, including Google’s Gemini, OpenAI’s ChatGPT, and Apple Intelligence Writing Tools were used in the preparation of this manuscript. These tools were used to edit text and included the use of the following prompts: “Make concise,” “Make concise while preserving figure callouts.” Additionally, ChatGPT was used to help generate alt text for figures using the text from figure legends as input alongside the Oxford University Press Alt Text guide. All text output from these models was subsequently edited and confirmed for scientific accuracy by an author.

## Results

### Effect of antibiotic treatment on bacterial load, diversity, and community composition

To evaluate the impact of antibiotic treatment on bacterial load, diversity, and community composition, we treated C57BL/6 mice from Jackson Laboratory (JAX) with a simplified antibiotic cocktail of vancomycin, neomycin, and ampicillin for seven days. This regimen, shown by us and others to mediate robust depletion of the microbiota within three to seven days while being well-tolerated by recipients [10, 11], was administered to JAX recipient mice. C57BL/6 mice from Taconic (TAC), which are well-established to harbor distinct enteric microbial communities compared to JAX mice [12, 13], served as microbiota donors, whereas untreated JAX and TAC mice served as controls (Fig. S1A). Faecal samples were collected on days 0, 1, 3, and 7 for 16S rRNA gene qPCR and sequencing. At baseline (day 0), bacterial load was not significantly different between JAX and TAC mice (Fig. S1B, two-way ANOVA followed by Tukey’s HSD *post hoc* test, *P* > .05). By day 1, antibiotics significantly reduced bacterial load in JAX mice (two-way ANOVA followed by Tukey’s HSD *post hoc* test, *P* < .05), with 16S rRNA gene levels falling below the limit of detection by day 7 (Fig. S1B).

Initial species (grouped at >97% sequence identity) richness was comparable between JAX and TAC mice (Kruskal–Wallis followed by Bonferroni’s correction, *P* > .05, mean ± SE: TAC = 80 ± 6 species, JAX = 66 ± 5; Fig. S1C). Antibiotic treatment significantly reduced species richness within one day compared to JAX controls (Kruskal–Wallis followed by Bonferroni’s correction, *P* < .05; mean ± SE: 43 ± 6 and 117 ± 2, respectively), with richness dropping to approximately one-sixth of the original level by day 7 (Fig. S1C, 9 ± 1), although sequencing suggested some bacteria persisted at low levels. To assess changes in community composition, phylogenetically aware unweighted UniFrac distances were calculated and visualized using PCoA. PCoA axis 1 explained 24.6% of the total variance, primarily reflecting antibiotic-induced shifts in JAX mice, whereas axis 2 (13.0%) separated untreated JAX and TAC samples (Fig. S1D). Antibiotic treatment and source explain large, highly significant shifts in community composition when including samples collected on days 0 and 7, before and after antibiotic treatment (PERMANOVA implemented using adonis2; Antibiotics: *R*^2^ ≈ 0.30, *F* = 81.7; Source: *R*^2^ ≈ 0.17, *F* = 45.2; both *P* < 1e-5; permutations were restricted within experiments). Between days 0 and 7 there is no detectable main effect of time (*R*^2^ ≈ 0.004, *P* = .385) nor Source×Day interaction (*R*^2^ ≈ 0.005, *P* = .204), suggesting temporal change in this window is minimal relative to treatment/source differences. These results demonstrate that one week of vancomycin, neomycin, and ampicillin treatment is sufficient to drastically reduce bacterial load and diversity and significantly alter gut microbiota composition.

### Transfer method regulates community assembly following antibiotic treatment

**Figure 1:**
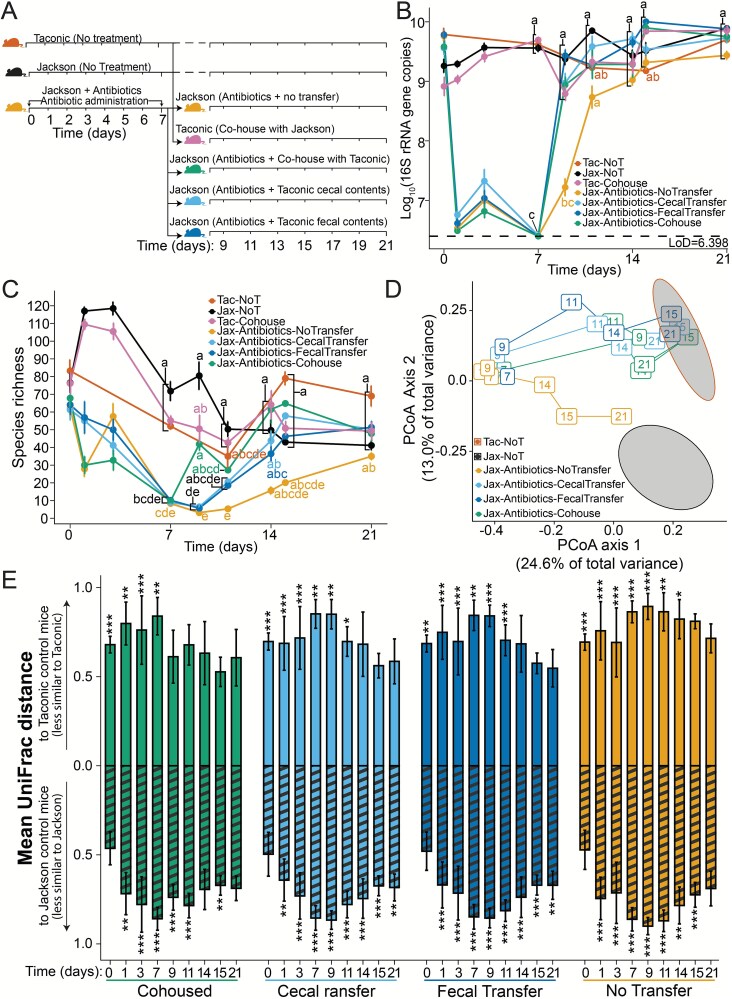
Co-housing of mice reconstitutes the bacterial microbiota more rapidly than faecal or caecal transplant. (**A**) Jackson mice were treated for one week with an antibiotic cocktail consisting of vancomycin, neomycin, and ampicillin in their drinking water. Antibiotic-treated Jackson mice were either allowed to recover naturally (Jax-Antibiotics-NoTransfer; *n =* 12, 8–12 samples per timepoint across three experiments), or were colonized with Taconic-associated microbes via gavage of caecal (Jax-Antibiotics-CaecalTransfer; *n =* 12, 7–12 samples per timepoint) or faecal (Jax-Antibiotics-FaecalTransfer; *n =* 12, 8–12 samples per timepoint) material, or co-housing (Jax-Antibiotics-Cohouse; *n =* 11, 6–11 samples per timepoint). Jackson (Jax-NoT) and Taconic (Tac-NoT) mice that did not receive antibiotics were maintained as controls. **(B)** Bacterial load measured by qPCR of the 16S rRNA gene throughout the final two weeks of the experiment shows the rapid recovery of bacterial load following microbiota transfer. The letters next to each point indicate significant groupings, those with the same letter were not significantly different from one another by two-way ANOVA followed by Tukey’s HSD *post hoc* test for pairwise comparisons with an alpha of 0.05 (**C**) bacterial species richness, the number of unique bacterial species observed per mouse, throughout the first week of the experiment via V4-16S rRNA gene sequencing. The letters next to each point indicate significant groupings, those with the same letter were not significantly different from one by Kruskal–Wallis followed by Bonferroni’s correction for multiple comparisons with an alpha threshold of 0.05. Compact letter display was condensed further for treatment groups sharing the same letter grouping on a given day, these are connected with a square bracket with letters listed in black, otherwise the color of the letters matches their respective treatment group. (**D**) Principal coordinate analysis of unweighted UniFrac distances calculated from species counts across all three weeks of the experiment illustrate the change in community composition following antibiotic treatment and microbiota transfer. Ellipses (95% confidence) represent the baseline faecal bacterial communities of untreated Jackson (Jax-NoT) and Taconic (Tac-NoT) mice during the first week of the experiment. (**E**) Bar heights indicate the mean unweighted UniFrac distance was calculated between the faecal bacterial community of Jackson mice treated with antibiotics that received microbial transfer via co-housing, caecal transfer, faecal transfer, or no transfer, and the faecal bacterial community of control Jackson and Taconic mice that never received antibiotics, pooled by week (days pooled: [0,7), [7,14), [14, 21]). Striped bars below the x-axis indicate the distance from untreated Jackson controls whereas solid bars above the x-axis indicate the distance from untreated Taconic controls. Error bars indicate the standard deviation of all pairwise comparisons between the indicated groups. A PERMANOVA test was performed on the unweighted UniFrac distance matrix for the comparisons shown followed by Bonferroni’s corrections for multiple comparisons (^*^P < .05, ^**^P < .01, ^*^^**^P < .001).

We compared the outcomes of different methods for transferring TAC microbiota into antibiotic-treated JAX mice. Antibiotics were stopped on day 7, and mice were either maintained without intentional microbial transfer, gavaged on day 8 and 9 with prepared caecal material or faecal material or co-housed on day 8 with untreated TAC donors for two weeks (Fig. 1A). TAC and JAX mice that did not receive antibiotics were also monitored over the same period.

Bacterial load increased significantly by day 9 in all transfer-recipient mice, regardless of method, compared to non-recipient mice (two-way ANOVA followed by Tukey’s HSD *post hoc* test, *P* < .05; Fig. 1B). Although non-recipient mice also showed detectable bacterial levels by day 9, their loads were nearly two orders of magnitude lower than those of recipient or untreated control mice. By day 11, 16S rRNA gene copy numbers were comparable across all groups, and by day 14, bacterial loads remained similar for the rest of the experiment (two-way ANOVA followed by Tukey’s HSD *post hoc* test, *P* > .05). In contrast, recovery of bacterial alpha-diversity progressed more slowly than bacterial load, particularly for the gavage-based transplantation methods (Fig. 1C). Although not statistically significantly different, bacterial richness decreased in untreated Jackson controls over the course of the experiment (Fig. 1C, means ± SE, day 0 = 76.5 ± 5.7; day 21 = 41.1 ± 2.7).

The effect of the transfer method on community composition was visualized using PCoA of unweighted UniFrac distances (Fig. 1D). Bacterial communities of antibiotic-treated JAX mice administered faecal or caecal transplants gradually approached the TAC donor community composition between days 9 and 11, converging by day 15 (Fig. 1D). Although faecal or caecal transplant restored bacterial loads to TAC donor levels within one day (Fig. 1B), the community composition was not fully recapitulated by day 9. In contrast, co-housed mice exhibited bacterial compositions equivalent to TAC donors after only one day (PERMANOVA of unweighted UniFrac distances followed by Bonferroni’s corrections; *P* > .05 on day 9; Fig. 1D and E). JAX mice that received antibiotics, but no transfer gradually returned towards untreated JAX mice, achieving a similar state to the original microbiota by day 21.

The unweighted UniFrac distances of treatment arms compared to untreated Taconic and Jackson control mice were analyzed using PERMANOVA. To account for temporal community composition drift and low sample sizes in untreated Taconic controls, daily treatment arm samples were compared to pooled control samples within the same week. Antibiotic-treated animals differed significantly from JAX controls after 1 day, with this difference persisting until day 7 (PERMANOVA of unweighted UniFrac distances followed by Bonferroni’s corrections; *P* < .001, see figure for *P* values associated with each group and day, Fig. 1E). The bacterial community of all groups differed from TAC controls, increasing until day 7 (PERMANOVA of unweighted UniFrac distances followed by Bonferroni’s corrections; *P* < .001; Fig. 1D and E). By day 9, antibiotic-treated JAX mice co-housed with TAC donors were no longer significantly different from TAC controls. Caecal or faecal transfer recipients differed from Taconic donors until day 14, suggesting varying transfer efficiency. Consistent with their bacterial loads (Fig. 1B), antibiotic-treated JAX mice recovered and were not significantly different from JAX controls by day 21 (PERMANOVA of unweighted UniFrac distances followed by Bonferroni’s corrections; *P* > .05 on day 21; Fig. 1D and E). However, the average distance between these groups remained elevated (mean ± SD: 0.69 ± 0.1) and had not returned to baseline (mean ± SD: 0.47 ± 0.1). Co-housed mice showed the fastest transition to a TAC-like community but were not significantly different from JAX controls on days 14 and 21, though different on day 15. This may indicate incomplete microbial transfer, JAX bacterial recovery in co-housed mice resulting in an intermediate community state or reflect ongoing temporal changes in the JAX and TAC control communities.

### Limited bacterial persistence following antibiotic treatment

**Figure 2:**
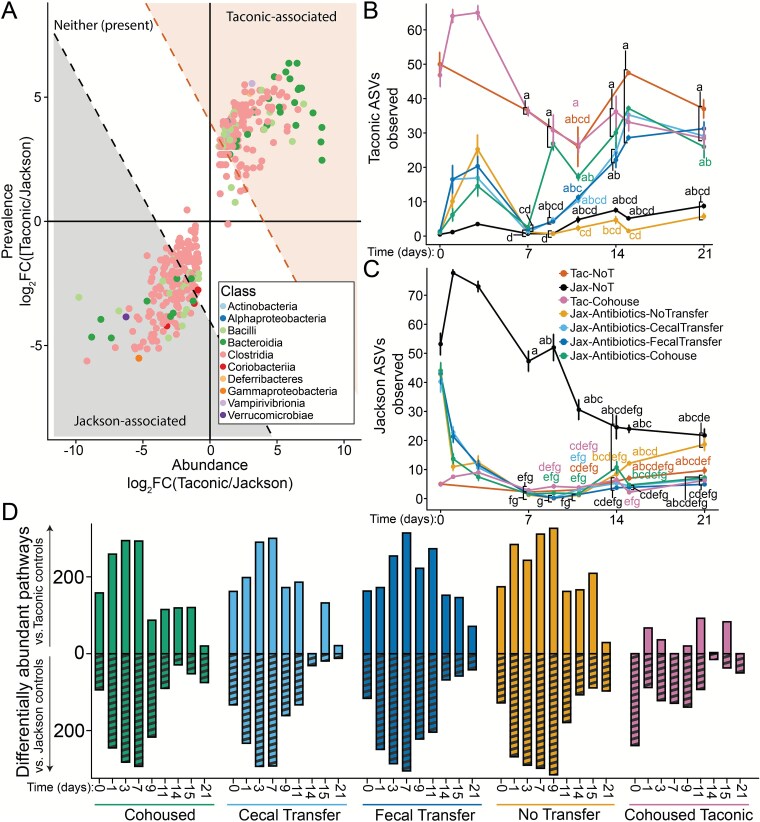
Defining and tracking Taconic-associated ASVs and their predicted functional capacities. (**A**) Bacterial ASVs were defined as being TAC or JAX associated based on their prevalence and abundance in samples collected from antibiotics-free JAX (*n =* 81) and TAC (*n =* 50) mice over the first week of the experiment. ASVs that had an absolute combined log_2_(fold change) > 4 were consider Taconic-associated whereas those that had an absolute combined log_2_(fold change) < 4 were considered Jackson-associated. Richness of (**B**) Taconic and (**C**) Jackson ASVs was monitored throughout the course of the experiment, plot show mean richness in each group at a given timepoint, error bars represent the standard error, compact letter display indicates results of pairwise Kruskal–Wallis followed by Bonferroni’s correction for multiple comparisons with an alpha threshold of 0.05. (**D**) Abundance of functional pathways were predicted using PICRUSt2. Bar heights indicate the number of pathways determined to be significantly differentially abundant using DESeq2 (alpha = 0.05) to compare between the faecal bacterial community of Jackson mice treated with antibiotics that received microbial transfer via co-housing, caecal transfer, faecal transfer, or no transfer, and Taconic co-housed mice, and the faecal bacterial community of control Jackson and Taconic mice that never received antibiotics, pooled by week (days pooled: [0,7), [7,14), [14, 21]). Tables detailing differential pathways for each timepoint and comparison are available in the Zenodo repository (10.5281/zenodo.15191166).

To understand bacterial persistence and transfer dynamics, ASVs were classified as TAC- (162 ASVs) or JAX-associated (124 ASVs), based on their prevalence in untreated mice in samples collected during the first week of the experiment (50 TAC and 81 JAX samples) (Fig. 2A). Additionally, ASVs that were not significantly associated with either source community were further subclassified as Neither (early/present) if they were present in untreated controls but not significantly associated with either group, or Neither (absent) if they were not observed in any of the controls in the first week. These subsets were tracked throughout the experiment.

The majority of ASVs observed in antibiotic treated mice on day 7 were classified as Neither (absent) (98 ASVs, Fig. S2A). Of the remaining ASVs observed in these mice, 22 were JAX-associated, 23 were TAC-associated, and 14 were present in control mice but not significantly associated with either vendor. We next compared bacterial species abundance in JAX antibiotic treated mice to untreated mice over the first week of the experiment using ANCOM-BC2 (Fig. S2B). On day 0, only one bacterial species (within the *Clostridia UCG-014* order) was significantly enriched in mice prior to receiving antibiotic treatment, suggesting these starting communities were relatively similar, as expected. After one day of antibiotic treatment, numerous species were differentially abundant between the two groups. Control mice were enriched for a diverse collection of bacterial species, whereas antibiotic treated mice were primarily enriched for *Lachnospiraceae* and *Clostridia* species, particularly from the *Clostridia vadin BB60* order. Species from the genus *Lachnospiraceae NK4A136* and order *Clostridia UCG-014* were enriched in both groups, highlighting species or strain specific responses to antibiotic treatment. On day 3, numerous *Bacteroidales* were enriched in antibiotic treated mice (e.g. *Muribaculaceae*, *Alistipes*, *Prevotellaceae UCG-001*), along with other species including various *Lachnospiraceae*. By day 7, only one *Staphylococcaceae* and one *Streptococcus* species were significantly enriched in antibiotic treated mice.

### Co-housing rapidly transfers donor-associated bacterial communities and their predicted functional capabilities

TAC ASVs were higher in TAC mice than in untreated JAX mice, significantly so on days 7 and 9 (Kruskal–Wallis followed by Bonferroni’s correction, *P* < .05; Fig. 2B). TAC and JAX ASVs decreased in richness from day 0 through day 9 in TAC and JAX control mice, with Taconic ASVs rebounding on subsequent days (Fig. 2B and C). Although this highlights temporal variation and oscillation in species richness, these differences were not statistically significant across timepoints sampled in our study (Kruskal–Wallis followed by Bonferroni’s correction, *P* > .05). Although there was a transient increase in the richness of TAC ASVs on days 1 and 3 in all groups measured, by day 7 the TAC-associated ASV richness in all JAX mice (mean = 0.7 – 2.6 Taconic ASVs per sample) was significantly lower than in TAC donors (~36.3 Taconic ASVs per sample) (Kruskal–Wallis followed by Bonferroni’s correction, *P* < .05; Fig. 2B). By day 9, TAC ASVs increased in co-housed mice (mean ± SE = 26.9 ± 2.0) and were higher than untreated JAX controls (mean ± SE = 0.7 ± 0.1) but not significantly different from TAC co-housed donors (mean ± SE = 30.8 ± 4.5; Kruskal–Wallis followed by Bonferroni’s correction, *P* > .05). At day 9, caecal or faecal transplants did not significantly increase TAC-associated ASV richness (means ± SEs = 4.6 ± 0.8 and 4.3 ± 0.4, respectively; Kruskal–Wallis followed by Bonferroni’s correction, *P* > .05). By day 15, the richness of TAC-associated ASVs in JAX mice that received antibiotics followed by caecal or faecal transplant was elevated relative to Jackson control mice and remained relatively steady until the end of the experiment on day 21. The level of JAX ASVs was low in all mice sourced from TAC, and JAX mice that received antibiotics followed by microbial transfer; additionally, there was no appreciable transfer of JAX microbes into TAC co-housed mice (Fig. 2C). Co-housing introduced donor-specific bacteria more rapidly than caecal or faecal transplant into antibiotic treated mice.

Predicted functional capacities, based on 16S rRNA gene sequence profiles (PICRUSt2), of faecal bacterial communities from JAX and TAC control mice were compared over the first week of the experiment. Numerous predicted functions were differentially abundant between vendors (Fig. S3A and B, Table S1). This included an enrichment of predicted “enterobactin biosynthesis,” “L-methionine salvage cycle III,” and glyoxylate cycle/bypass genes in JAX mice (Fig. S3B). TAC mice were enriched for various predicted fatty acid biosynthetic pathways (e.g. palmitate, oleate, and stearate) as well as ubiquinol biosynthesis. We next compared PICRUSt2 predicted functional capacities of antibiotic-treated JAX mice (split by method of transfer) and TAC co-housed donors to JAX and TAC controls grouped by week to identify differentially abundant pathways using DESeq2 (Fig. 2D) [69]. A larger number of differentially abundant pathways indicates greater functional disparity. Consistent with our taxonomic results, the number of differentially abundant pathways between co-housed JAX mice and TAC control mice dropped off rapidly, from over 200 on day 7 to less than 100 on day 9. Differential pathways for each comparison and timepoint are available in output tables on Zenodo (10.5281/zenodo.15191166). Mice receiving a caecal transplant did not drop below 100 differential pathways until day 14, while faecal transplant recipients did not until day 21. Co-housing led to a more rapid and comprehensive transfer of Taconic-associated bacterial taxa and their predicted functional capabilities compared to caecal or faecal transplantation.

### Transfer of specific bacterial taxa differs between methods

**Figure 3:**
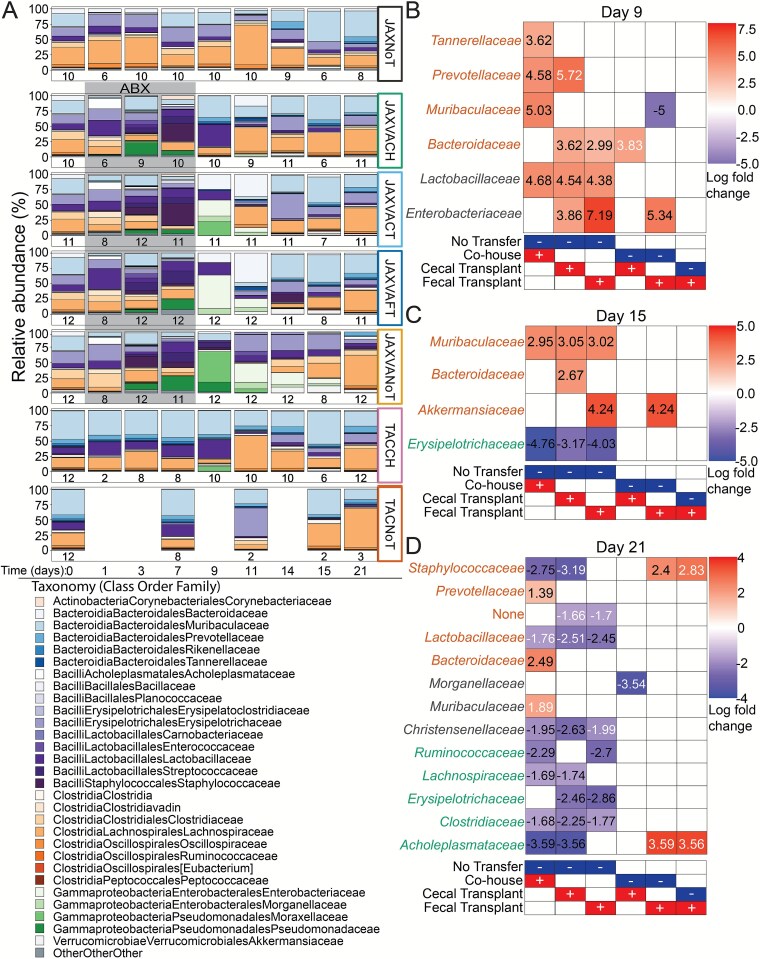
Specific bacterial taxa are enriched across transfer methods. **(A**) Mean proportional relative abundance of bacterial families throughout the course of the experiment in each of the treatment groups, averaged across all samples on each day. Grey background indicates samples collected in from mice that had been provided antibiotics in their drinking water. The number under each bar indicates the number of samples for each group and timepoint following filtering. (**B–D**) Bacterial families associated with original bacterial communities of mice from Taconic (orange), Jackson (green) or neither/both (black) that were significantly enriched or depleted for the comparisons shown below each plot as determined using ANCOM-BC2. Panels show results from days 9 **(B)**, 15 **(C)**, and 21 **(D)**. Log fold change values in black remained significant after applying a stringent mixed directional FDR control. The panel underneath each heatmap indicates the groups being compared in each column. The red group (+) was the numerator, and the blue group (−) was the denominator when calculating fold change. Positive fold change values are shaded red in the heat map and negative fold change values are shaded in blue, indicating which of the two groups being compared in a given column each bacterial family, displayed on rows, was enriched in.

Changes in bacterial community composition were profiled throughout the experiment (Fig. 3A). The relative abundance of *Bacilli* and *Gammaproteobacteria* increased in mice receiving the antibiotic cocktail by day 3 (Fig. 3A), though the overall bacterial load and richness remained low (Fig. 1B and C). By day 9, co-housed mice were significantly enriched for TAC ASVs from the *Tannerellaceae*, *Prevotellaceae*, and *Muribaculaceae* families, and *Lactobacillaceae* ASVs that were not associated with either supplier, relative to mice without transfer (ANCOM-BC2, *P* < .05; Fig. 3B). Mice receiving caecal or faecal transplants were also enriched for these *Lactobacillaceae* ASVs, along with un-associated *Enterobacteriaceae* ASVs (Fig. 3B). *Bacteroidaceae* ASVs associated with the original TAC communities were enriched in mice receiving faecal or caecal transplants (Fig. 3B). *Enterobacteriaceae* ASVs were significantly enriched in mice receiving caecal transplant compared to co-housed mice, whereas co-housed mice were enriched for *Muribaculaceae* ASVs.

TAC-associated ASVs from the *Muribaculaceae* family were significantly enriched in all mice receiving a microbiota transfer at day 15, regardless of method (Fig. 3C). TAC *Bacteroidaceae* ASVs were enriched in mice receiving a caecal transplant, whereas TAC-associated *Akkermansiaceae* ASVs were enriched in mice receiving a faecal transplant compared to the no transfer and co-housed groups. JAX-associated *Erysipelotrichaceae* ASVs were the first to recover in antibiotic-treated mice without a transfer and were significantly enriched by day 15 compared to co-housed mice or mice receiving a faecal or caecal transplant (Fig. 3C).

By day 21, significant differences in bacterial taxa enrichment were observed across groups (Fig. 3D). TAC *Staphylococcaceae* ASVs were enriched in the no transfer and faecal transplant groups compared to co-housed and caecal transplant recipient mice, whereas TAC *Lactobacillaceae* ASVs were enriched in the no transfer group compared to the transfer-recipient groups. *Christensenellaceae* ASVs, not associated with either supplier, were enriched in the no transfer group compared to all transfer groups. Several JAX-associated taxa, including *Ruminococcaceae*, *Lachnospiraceae*, *Erysipelotrichaceae*, and *Clostridiaceae*, were enriched in the no transfer group compared to all transfer groups. The faecal transplant groups exhibited distinct enrichment patterns, including higher levels of TAC-associated *Staphylococcaceae* and JAX-associated *Acholeplasmataceae* compared to co-housed or caecal transplant recipient mice. JAX *Acholeplasmataceae* were also enriched in the no transfer group compared to co-housed or caecal transplant recipient mice. Distinct transfer methods differentially enriched specific bacterial taxa over time, with co-housing favoring *Muribaculaceae* and certain donor-associated lineages earlier, whereas faecal and caecal transplants more strongly enriched for *Bacteroidaceae*, *Enterobacteriaceae*, and *Akkermansiaceae*; mice that did not receive a transfer exhibited recovery of native JAX-associated taxa and unique enrichments not observed in other groups by day 21.

### Bacterial diversity and community composition differ across gastrointestinal sites and method of transfer

**Figure 4:**
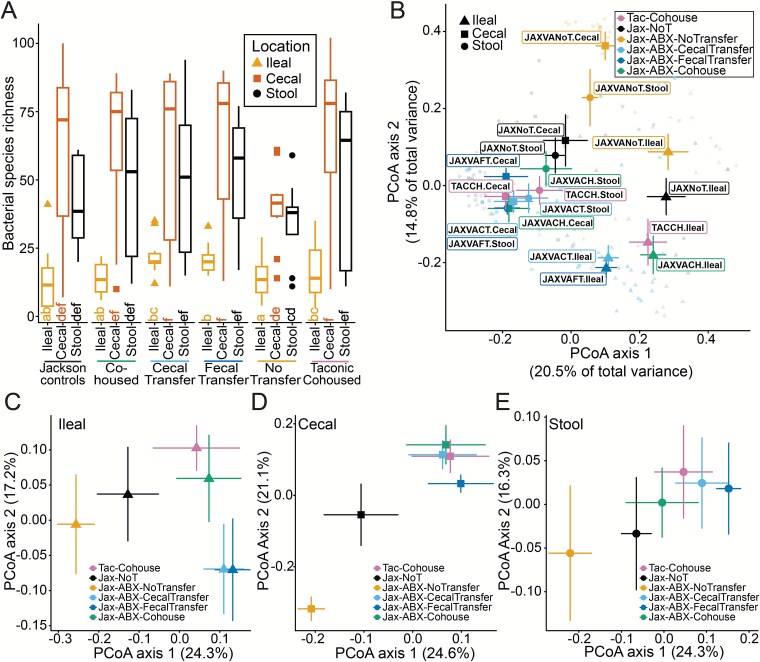
Bacterial diversity and community composition across different body sites and treatment groups. (**A**) Bacterial species richness, the number of bacterial species observed in caecal, ileal, and stool samples across different groups, including Jackson controls, co-housed, caecal transfer, faecal transfer, no transfer, and Taconic co-housed mice. The letters under each bar indicate significant groupings, those with the same letter were not significantly different from one another. Observed richness was compared using a linear mixed-effects model with sequencing library as a random effect, post hoc comparison of marginal means were Tukey-adjusted; significance groups are indicated by differing letters (α = 0.05). Colors and shapes indicate gastrointestinal site where sample was collected. (**B**) Plot of principal coordinate analysis (PCoA) of unweighted UniFrac distances illustrating bacterial community composition from caecal, ileal, and stool samples, across each method of transfer, including Jackson controls and co-housed Taconic donors. PCoA of unweighted UniFrac distances of samples on Day 21 across treatment groups from (**C**) ileal contents, (**D**) caecal contents, or (**E**) stool. In (**B-E**) shapes indicate the gastrointestinal site where samples were collected, and colors indicate the treatment group.

Bacterial diversity and community composition varied across gastrointestinal sites and treatment groups. Lower richness was consistently observed in ileal contents compared to caecal contents and stool, as well as at all sites in mice without microbiota transfer after antibiotic treatment (Fig. 4A). Stool richness trended higher in transfer-recipient mice than JAX controls but lower than TAC controls, suggesting incomplete bacterial taxa transfer. Species richness was significantly lower in antibiotic treated JAX mice with no transfer compared to mice that received a faecal or caecal transplant across all gastrointestinal sites (Fig. 4A; linear mixed-effects model with sequencing library as a random effect, post hoc comparison of marginal means was Tukey-adjusted, *P* < .05). Richness was lower, but not significantly so, in no transfer mice compared to co-housed mice. PCoA illustrated community composition differences, with samples clustering according to treatment and site (Fig. 4B). Samples from JAX mice treated with antibiotics but no transfer clustered together, away from samples from transfer-recipient mice and control communities, with samples from the same gastrointestinal site clustering together. To compare community compositions across treatments, PCoA was performed on samples subset by the gastrointestinal site (Fig. 4C–E). Ileal, caecal, and stool communities of JAX co-housed mice were closely aligned with TAC donor mice, whereas ileal communities of JAX faecal or caecal transplantation mice clustered separately (Fig. 4C), a difference that was not as pronounced in caecal or stool samples (Fig. 4D and E). These findings provide insights into the dynamics of bacterial community re-establishment across microbiota transfer methods.

### Viral community is depleted post-antibiotics

We examined the viral response to antibiotic treatment, noting a significant reduction in bacterial load and diversity over the first week (Fig. 1B and C). Short-read shotgun sequencing of DNA from VLPs assessed the impact of antibiotics on viral communities. Viral richness (unique contigs from sequencing data) was compared from days 0 to 7 between TAC and JAX controls and antibiotic-treated JAX mice. Read counts were normalized to reduce library batch effects. Viral richness dropped significantly from 596 ± 84 (mean ± SE) unique viral contigs on day 0 to 34 ± 2 on day 7 (Fig. S4A). This pattern persisted when contigs with clear viral signatures were analyzed and when aggregated into clusters representing viral species, genera, and families (Fig. S4A). The reduction in viral richness indicates viral populations’ susceptibility to bacterial community disruptions, likely due to their dependence on bacterial hosts. A shift in viral community composition, primarily driven by antibiotic treatment, was observed in PCoA of Jaccard distances (Fig. S4B). The separation along axis 1 was mainly between antibiotic-treated and untreated groups, highlighting antibiotics’ substantial impact on viral community composition. Similar results were found when considering only high-quality viral contigs or different taxonomic ranks (Fig. S4B).

### Co-housing facilitates more rapid transfer of donor viral communities

**Figure 5:**
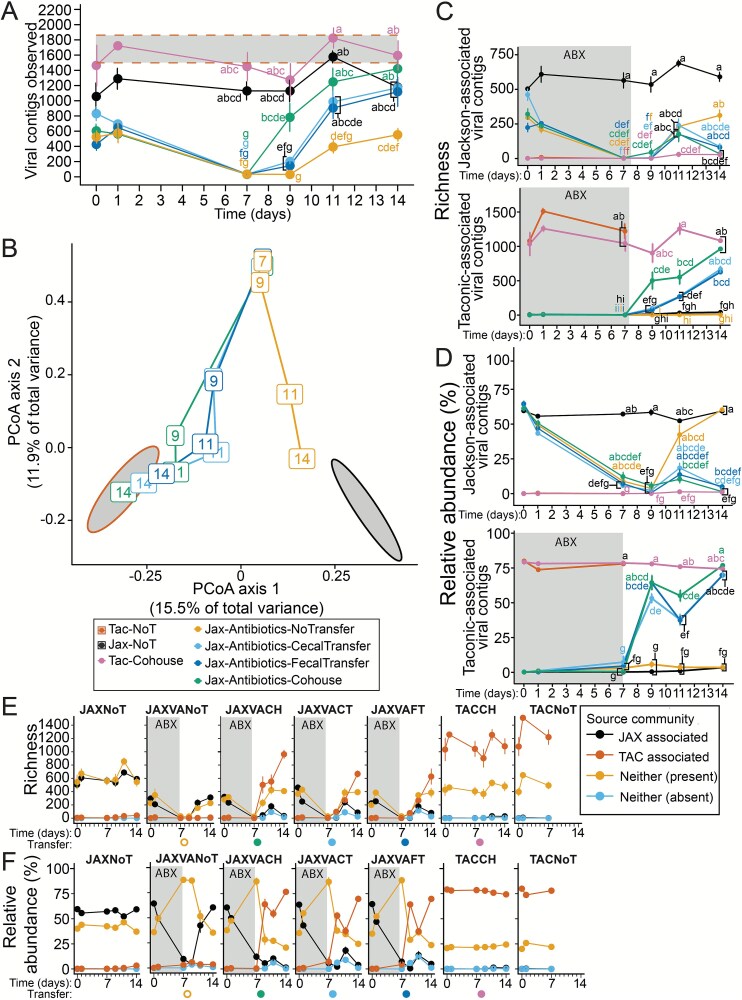
Co-housing of mice reconstitutes the viral microbiota more rapidly than faecal or caecal transplant. (**A)** Viral richness from days 0 through 14 of the experiment shows the rapid recovery of viral diversity following transfer. The letters next to each point indicate significant groupings, those with the same letter were not significantly different from one another by pairwise Kruskal–Wallis followed by Bonferroni’s correction for multiple comparisons with an alpha threshold of 0.05. Only days 7-14 were compared in statistical analyses displayed in this figure. (**B**) Principal coordinate analysis of Jaccard distances calculated from contig read counts across two weeks of the experiment illustrate the change in community composition following antibiotic treatment and microbiota transfer. Ellipses (95% confidence) represent the baseline faecal viral communities of untreated Jackson (Jax-NoT) and Taconic (Tac-NoT) mice over the first week of the experiment. (**C**) Richness and (**D**) relative abundance of viral contigs defined as (i) Jackson or (ii) Taconic-associated throughout the course of the experiment. The letters next to each point indicate significant groupings, those with the same letter were not significantly different from one by pairwise Kruskal–Wallis followed by Bonferroni’s correction for multiple comparisons with an alpha threshold of 0.05. The (**E**) richness and (**F**) relative abundance of viral contigs associated with mice from Jackson, Taconic, or neither/both, over the first two weeks of the experiment. *n =* 6–11 samples per treatment group and timepoint, for all groups excluding TACNoT controls (*n =* 1–5).

To evaluate the effects of different microbiota transfer methods on viral communities in antibiotic-treated mice, we compared viral richness and community composition post-transfer. Samples were collected from days 7 to 14 to observe viral population reestablishment. This analysis included all contigs from short read sequencing of VLPs, avoiding biases from viral annotation and prediction algorithms. Viral richness, measured by unique viral contigs, did not uniformly increase across all groups after antibiotics ceased. By day 9, co-housed JAX mice showed a quicker rise in viral richness than other groups, with no significant difference from co-housed TAC donor mice (Kruskal–Wallis followed by Bonferroni’s correction, *P* > .05; Fig. 5A). Viral richness was low in mice with faecal or caecal transfers, or no transfer, at this point. By day 11, viral richness increased in all groups, and even though transfer-receiving groups had lower richness than Taconic donors, the difference was not significant. Antibiotic-treated mice without transfer had significantly lower viral richness, which only slightly increased by day 11. By day 14, viral richness in all transfer groups converged, similar to untreated controls, indicating successful re-colonization (Kruskal–Wallis followed by Bonferroni’s correction, *P* > .05).

We assessed the impact of transfer methods on viral community composition using PCoA of Jaccard distances. Results indicated a gradual alignment of viral communities in faecal and caecal transplanted mice with TAC donor profiles between days 9 and 11, achieving full convergence by day 14 (Fig. 5B). Co-housed mice showed rapid convergence with donor profiles by day 9, suggesting co-housing enhances efficient viral transfer. In contrast, antibiotic-treated mice without transfer experienced delayed recovery, remaining distinct from donor and control profiles until the final sampling day, gradually nearing the original JAX community by day 14.

These findings highlight the critical role of microbiota transfer in reestablishing viral communities. Co-housing was the most effective for rapid and complete viral transfer, while faecal and caecal transplants also supported successful colonization, though more slowly. Mice without transfer showed delayed and incomplete viral community recovery, emphasizing the need for external microbial inputs to restore gut virome diversity and composition post-antibiotic treatment.

### Transfer of Taconic-associated viruses to Jackson mice

To monitor virus movement between TAC and JAX mice, viral contigs were categorized as TAC, JAX, or neither/both, based on their prevalence and abundance in samples from the first week of the experiment (34 TAC and 50 JAX samples) without antibiotics. Out of 6249 contigs, 2325 were TAC-associated and 832 were JAX-associated. Antibiotic treatment significantly reduced the richness and relative abundance of JAX-associated viruses (Kruskal–Wallis followed by Bonferroni’s correction, *P* < .05; Fig. 5C–F). TAC and JAX control mice maintained relatively consistent viral richness and abundance (Kruskal–Wallis followed by Bonferroni’s correction, *P* > .05 across timepoints; Fig. 5C–F), with TAC mice having more unique viral genome contigs than JAX controls (Fig. 5C and E).

After microbiota transfer, TAC-associated virus richness and abundance in co-housed JAX mice was not significantly different from TAC donors by day 9 (Kruskal–Wallis followed by Bonferroni’s correction, *P* < .05; Fig. 5C and D). Conversely, Taconic viral richness was significantly in lower faecal and caecal transplant recipients on day 9 compared to Taconic donors (Kruskal–Wallis followed by Bonferroni’s correction, *P* < .05; Fig. 5C). By day 9, TAC-associated virus relative abundance in faecal and caecal transplant recipients was similar to donors, though still significantly lower (Fig. 5D). By day 11, three days after transplantation, TAC-associated virus abundance in faecal or caecal transplant recipients dropped well below donor levels (Fig. 5D and F). In contrast, co-housed mice maintained relatively stable TAC-associated virus abundance, although it was significantly lower than TAC co-housed donors on day 11. This decline was accompanied by a slight resurgence of JAX-associated viruses on day 11 (Fig. 5C–E), but this rebound was not sustained, except in JAX antibiotic-treated mice without transfer, where JAX-associated viruses persisted (Fig. 5D and F).

Despite only receiving two gavages of faecal or caecal contents, the number of unique viral genome fragments in transplanted mice increased, with detectable TAC-associated virus levels rising even after one week (Fig. 5C and E). However, TAC-associated virus richness in JAX mice with caecal or faecal transplants remained lower than TAC donors one week after gavage, on day 14 (Fig. 5C and E).

Viral richness in JAX mice treated with antibiotics and no transfer were low compared to Jackson control mice throughout the experiment (through day 14), including prior to treatment on Day 0 (Fig. 5A). This richness remained low despite JAX-associated virus relative abundance returning to untreated JAX control and day 0 levels by day 11 (Fig. 5C–F). The different baseline viral diversity in these groups of JAX mice highlight the potential for inter-cage variability in viral community composition.

These findings underscore the complexity of viral community dynamics post-microbiota transfer, with co-housing being the most effective method for rapidly restoring donor-associated viral communities. Although faecal and caecal transplants also facilitated donor virus colonization, the process was slower and less consistent when considering viral richness and abundance.

### Binning viral genomes confirms rapid microbial transfer following co-housing

**Figure 6:**
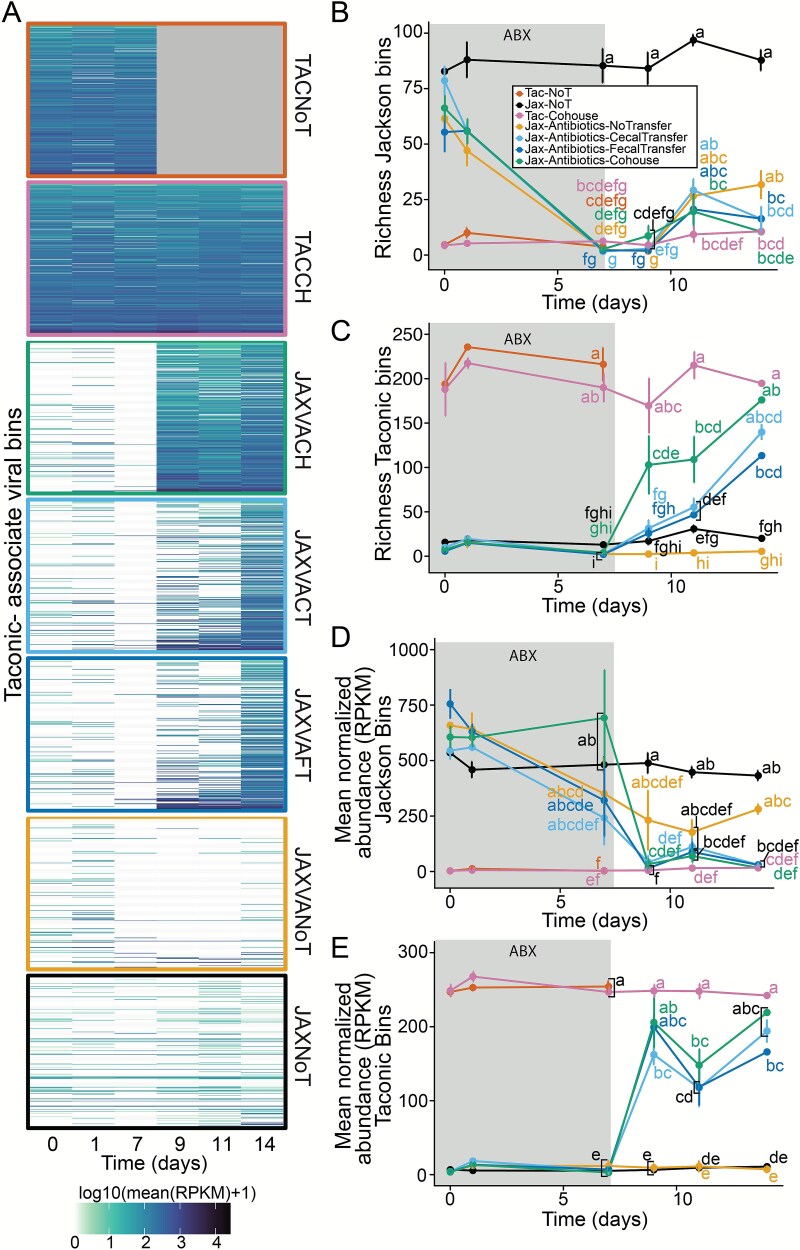
Binning viral genomes confirms rapid microbial transfer following co-housing. (**A)** Heatmap illustrating the richness of Taconic-associated viral bins over time in each treatment and control group as indicated on the right side of each panel. Line plots of the richness of (**B**) Jackson- and (**C**) Taconic-associated viral bins and mean normalized abundance of (**D**) Jackson- and (**E**) Taconic-associated viral bins over the course of the experiment. The letters next to each point indicate significant groupings, those with the same letter were not significantly different from one another by Kruskal–Wallis test followed by Bonferroni correction for multiple comparisons with an alpha of 0.05. *n =* 6–11 samples per treatment group and timepoint, for all groups excluding TACNoT controls (*n =* 5). Only samples collected on days 7–14 were statistically compared.

Contigs were binned into homogeneous groups representing individual viral genomes using CoCoNet [80], based on sequence composition and coverage variation. 1525 contigs were assigned to 629 unique bins (mean 2.4 contigs per bin, 426 bins with only one contig). Bins were categorized as JAX- or TAC-associated, or neither, according to previous contig classifications. No bins contained both JAX and TAC-associated contigs, allowing entire bins to be classified based on the presence of any TAC- or JAX-associated contigs (Fig. 6A–E). The richness of JAX-associated bins significantly decreased by day 7 in antibiotic-treated mice (Kruskal–Wallis followed by Bonferroni’s correction, *P* < .05; Fig. 6B). The richness of TAC-associated bins increased significantly on day 9 in co-housed mice (Kruskal–Wallis followed by Bonferroni’s correction, *P* < .05; Fig. 6A and C) but did not reach similar levels until day 14 in faecal or caecal transplant mice. Additionally, the average abundance of bins was quantified based on sequencing read depth, normalized by bin length and sample sequencing depth. The average abundance of JAX bins (RPKM) slightly decreased after antibiotic treatment and was significantly lower on day 9 in mice with microbiota transfer (Kruskal–Wallis followed by Bonferroni’s correction, *P* < .05; Fig. 6D). The average abundance of TAC bins sharply increased in all transfer-recipient mice on day 9 (Fig. 6E). These findings align with our annotation free contig-level analysis, showing a rapid increase in viral richness in co-housed mice compared to those with faecal or caecal transplants (Figs 5C and 6C). The average abundance (RPKM) of Taconic bins increased in all transfer groups by day 9, mirroring the increased relative abundance observed in the contig-based analysis (Figs 5D and 6E).

### Dynamics of bacteriophages predicted to target discriminatory bacterial taxa

**Figure 7:**
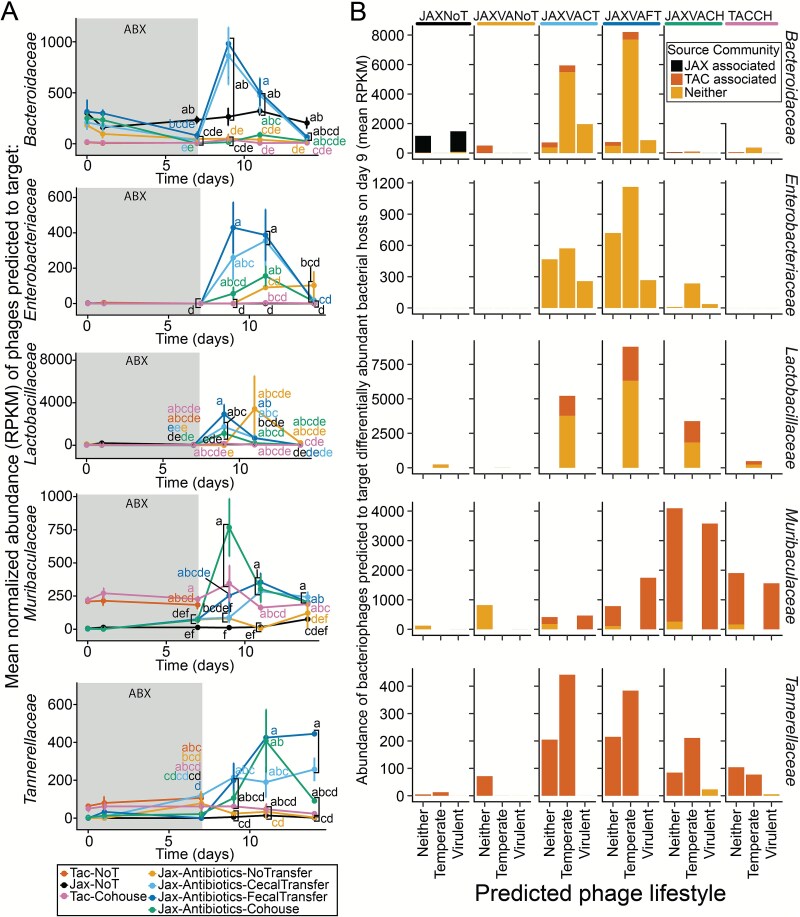
Abundance of bacteriophages mirrors discriminatory bacterial hosts. (**A**) The mean normalized reads per kilobase per million mapped reads (RPKM) abundance of phage bins predicted to target bacteria that were differentially abundant on day 9 (Fig. 3B) or day 15 (Fig. 3C), over time. Other bacterial families were either not predicted to be hosts for phages in this dataset or were not significantly different between any of the groups at the timepoints tested (Akkermansiaceae). The letters next to each point indicate significant groupings, those with the same letter were not significantly different from one another by Kruskal–Wallis test followed by Bonferroni correction for multiple comparisons with an alpha of 0.05. (**B**) Bar plots illustrating the abundance (mean RPKM) of phage bins targeting discriminatory bacterial taxa on day 9. Each column represents a treatment group. Within each plot, phages are split based on their predicted lifestyle. Rows are faceted by the family of the predicted bacterial host. *n =* 6–11 samples per treatment group and timepoint, for all groups excluding TACNoT controls (*n =* 5).

We examined the dynamics of phages targeting bacterial families with differential abundance during transfer. *Tannerellaceae*, *Prevotellaceae*, *Muribaculaceae*, *Bacteroidaceae*, *Lactobacillaceae*, *Enterobacteriaceae*, *Akkermansiaceae*, and *Erysipelotrichaceae* distinguished treatment groups at days 9 and 15 (Fig. 3B and C). Using iPHoP [78], we assessed the predicted bacterial host range for filtered phage contigs. No phages were predicted to target *Erysipelotrichaceae* or *Prevotellaceae*, and *Akkermansiaceae*-targeting phages were rare and not significantly different across groups (data not shown, available in Zenodo repository). The abundance of phages targeting the remaining bacterial families varied over time (Fig. 7A). Phages targeting *Bacteroidaceae* were elevated on day 9 in caecal and faecal transplant recipients, aligning with bacterial data (Fig. 3B). Most phages in transplant recipients were temperate and not specifically linked to the original JAX or TAC communities (Fig. 7B), with a few originally TAC-associated. JAX controls maintained JAX-associated phages targeting *Bacteroidaceae*, predicted to be virulent. Also mirroring the abundance of their bacterial hosts, *Enterobacteriaceae*-targeting phages peaked on day 9 in faecal transplant recipients (Fig. 7A and B). All *Lactobacillaceae*-hosted phages were temperate and increased on day 9 in transfer-recipient groups (Fig. 7A and B). *Muribaculaceae*-hosted phages were not temperate, with elevated abundance on day 9 in co-housed mice (Fig. 7A and B). Although many *Muribaculaceae*-targeting phages were predicted to be virulent, their abundance still matched their bacterial hosts (Fig. 3B). Despite *Tannerellaceae* bacterial enrichment on day 9 in co-housed mice, their targeting phages were not elevated. Phage abundance closely mirrored their predicted bacterial hosts, suggesting induction of temperate phages post faecal or caecal transplant, in contrast to transfer of virulent phage specifically targeting a high-abundance bacterial community member, *Muribaculaceae,* in the context of co-housing.

## Discussion

This study compared co-housing, faecal, and caecal transplantation in antibiotic-treated mice to reconstitute bacterial and viral communities [34, 35, 37, 46]. Transferring donor microbiota allows researchers to examine how composition influences health and disease. However, there is a lack of systematic comparisons of commonly used transfer methods. We addressed this gap by assessing the efficiency, dynamics, and ecological outcomes of three widely used techniques. Our findings are relevant not only for improving experimental methods and enhancing the reproducibility of mouse-based studies on host–microbe interactions but also provide insight into microbial community dynamics during and after antibiotic-based disturbances and following multiple forms of intervention post-disturbance.

Our study indicates that co-housing is the most effective method for rapid microbiota transfer, quickly reconstituting both bacterial and viral communities, aligning with previous studies showing that co-housing facilitates the transfer of donor-associated microbial taxa more robustly than gavage methods [36, 46]. Sustained microbial exchange through natural behaviours like coprophagy and direct contact during co-housing reflects ecological and social interactions in natural settings. In contrast, faecal or caecal transplantation homogenizes donor material in buffer and delivers a single bolus, which can disrupt spatial structure and expose strict anaerobes and phages to processing steps (e.g. air, temperature shifts) that may reduce viability. Co-housing supplies microbes *in situ* through repeated, lower-dose exposures via coprophagy, grooming, and shared cage surfaces, preserving native matrices (mucus, aggregates, biofilm fragments) and the spatial organization of the lumen community. This sustained, multiroute inoculation may increase propagule pressure and help taxa cross colonization thresholds. Repeated co-transfer of bacterial hosts with their phages may also promote stable coupling of viral and bacterial dynamics. Together, these features provide a potential explanation for the efficient reconstitution observed with co-housing relative to the bolus-based methods. Although the natural behaviours of mice limit our ability to translate findings directly to humans, the method-dependent reassembly dynamics we observed encourage investigation into post-antibiotic recolonization strategies and the resilience of these microbial communities in the face of widespread antibiotic usage in humans [81, 82].

Co-housed mice rapidly reconstitute microbial diversity and functionality, making co-housing useful for time-sensitive microbiota establishment. Faecal and caecal transplantation, though less efficient in the short term, achieved comparable outcomes by day 21. These methods may still be valuable in experimental scenarios where co-housing is impractical, but the slower pace of microbial establishment emphasizes the importance of considering time frames in experiments. Researchers should select transfer methods that align with study goals to optimize reproducibility and minimize variability.

Antibiotic cocktail treatment significantly reduced intestinal bacterial loads within a day, suggesting that shorter treatment duration from the currently commonly used 1-2 weeks before microbiota transfer may potentially be effective. Mice without transfer showed delayed and incomplete microbiota recovery, possibly because they have lost access to microbes that were eliminated by the antibiotics. Although bacterial load was below the limit of detection, and individual antibiotic treated mice had very few detectable bacterial species in their stool on day 7, alpha diversity increased gradually in this group without any experimental microbiota transfer. The profile of bacterial species that were enriched in antibiotic treated compared to control JAX mice shifted throughout the week of antibiotic treatment. A majority of the ASVs observed on day 7 of antibiotic treatment were not detected in JAX or TAC control mice, suggesting that antibiotic treatment allowed very low abundance or novel species to reach levels detectable via 16S rRNA gene amplicon sequencing. Species from the same genus or order displayed differential responses to antibiotic treatment highlighting the potential for species and strain specific responses to disturbances. *Clostridia vadin BB60* species showed early resistance to the antibiotic cocktail, in line with previous reports of persistence following antibiotic exposure for this taxa [83, 84]. One *Staphylococcaceae* and one *Streptococcus* species were in enriched on day 7. These are early colonizers of the neonatal murine gastrointestinal tract, as well as common colonizers of the adult female mouse skin and mucosa in animals housed on our campus [85]. This may reflect similar niche availability following broad antibiotic treatment as is observed in the initial period of neonatal gastrointestinal colonization, or potential reservoirs for these microbes that are not as impacted by oral antibiotics. Although JAX microbes were not readily detected on day 7, the community recovered, at least partially, to its original state. This suggests that native species persisted below the limit of detection following antibiotic treatment and were able to re-establish a (reduced) version of the original community structure once the selective pressure of antibiotics was removed. This limited natural recolonization highlights the potential for gut microbiota resiliency over the longer term. The study highlighted the interdependence of bacterial and viral dynamics, reflecting the ecological relationships between bacteriophages and their hosts. Antibiotics rapidly depleted bacteria, leading to a slightly delayed but robust reduction in phage communities. Co-housed mice quickly established donor-associated bacterial taxa like *Muribaculaceae* and *Tannerellaceae*, along with their phages. In contrast, early introduction of bacteria such as *Bacteroidaceae*, *Lactobacillaceae*, and *Enterobacteriaceae* via faecal or caecal transplant, was linked to temperate phages targeting these hosts, which were not always main members of the donor’s viral community. The early colonization by these taxa may reflect increased aerotolerance and colonization of uninhabited gastrointestinal tract niches. These phages likely emerged via induction from pioneering donor bacteria during early transfer and recolonization. The synchronized reconstitution seen with co-housing emphasizes the role of ecological interactions in microbial recovery, suggesting that co-housing may offer a more natural environment for community establishment.

The site-specific variation in microbial recovery across gastrointestinal regions underscores the complexity of microbiota reconstitution and the selective pressures shaping biogeography in the gut including variation in oxygen content, pH, and mucosal architecture [86]. When analyzed within sites, ileal communities of co-housed mice converged toward TAC mice, whereas ileal communities after faecal or caecal bolus transplantation remained distinct; this difference was attenuated in caecal and stool samples. The ileum may require repeated seeding to overcome washout and to colonize mucosal niches, whereas a single bolus more readily takes hold in the caecum and distal gut. The distinct microbial diversity and composition between ileal, caecal, and faecal samples emphasize the importance of sampling multiple regions to capture microbiota dynamics. Additionally, these patterns have practical consequences: stool-only sampling can overestimate whole-gut reconstitution; transfer modality may matter most for small-intestine outcomes; and interventions aimed at upper gut recovery may need repeated dosing, protected formulations, or substrate support to enable stable engraftment. These findings align with previous work showing anatomical differences in microbial communities and their functions [87–90].

Systematic evaluations of transfer methods across different contexts are crucial. Future studies should investigate how donor-recipient compatibility, microbial complexity, and host genotype influence outcomes. Although this study focused on bacterial and viral communities, incorporating fungi and archaea will provide a more comprehensive understanding. Additionally, only female mice were used in this study which facilitated co-housing in these experiments. However, the lack of males is a limitation of this study given sex differences in immunity and endocrine status [91, 92]. Additionally, the role of cross-sex microbial transfer was not addressed but is relevant when considering microbial exchange in natural populations or assessing donor-recipient compatibility during microbiota transplantation. Additionally, our functional profiles were based on predictions generated by PICRUSt2 from 16S rRNA gene amplicon sequence profiles and is a limitation of the study. Advancements in functional and longitudinal metagenomic analyses, coupled with improved animal models, will enhance our ability to study microbiota establishment and its implications for host health and disease. As we expand comparisons to new donor/recipient sources and microbial compartments, our comprehensive virome profiling pipeline, utilizing contig-based, classification-agnostic, and robust binning and classification methods, will ensure consistent capture of even poorly characterized or emerging viral populations across different experimental models. Characterization of human viral communities has broadly lagged the study of bacterial communities, the virome of experimental mice remains even more enigmatic. Our method successfully facilitated virome analysis and comparisons, and it could also be extended to analyze other poorly annotated microbes like fungi and archaea.

This study systematically compared microbiota transfer methods, refining experimental approaches in microbiome research and revealing ecological and functional dynamics underlying microbial reconstitution. These insights will facilitate tailoring of transfer methods to individual studies, thereby enhancing reproducibility and translational potential, and advancing our understanding of the microbiome’s role in health and disease.

## Supplementary Material

Figure_S1_2025_11_06_wraf256

Figure_S2_2025_11_06_wraf256

Figure_S3_2025_11_06_wraf256

Figure_S4_2025_11_06_wraf256

Supplementary_Figure_Legends_wraf256

Table_S1_2025_10_13_wraf256

## Data and materials availability

The data from this study are tabulated in the main paper and supplementary materials. All reagents are available from M.T.B. under a material transfer agreement with Washington University. Additionally, all sequencing data generated in this study, including 16S rRNA gene amplicon data and virome shotgun metagenomic data, have been deposited in the European Nucleotide Archive (ENA), under accession number PRJEB102596. Data files, metadata, and scripts used for analysis are available at Zenodo (https://doi.org/10.5281/zenodo.15191166) and will be made publicly accessible upon publication. Mapping between raw files submitted to ENA all relevant metadata are available at Zenodo (https://doi.org/10.5281/zenodo.15191166).

## Code availability statement

Scripts and code used for analysis are available on Zenodo (https://doi.org/10.5281/zenodo.15191166) and will be made publicly accessible upon publication.
