# Supplementary figures and images for: Differential microbial community assembly following co-housing versus microbiota transplant
Source: ISME J. 2025 Nov 17;19(1):wraf256. doi: 10.1093/ismejo/wraf256 (PMC12694414; doi:10.1093/ismejo/wraf256)

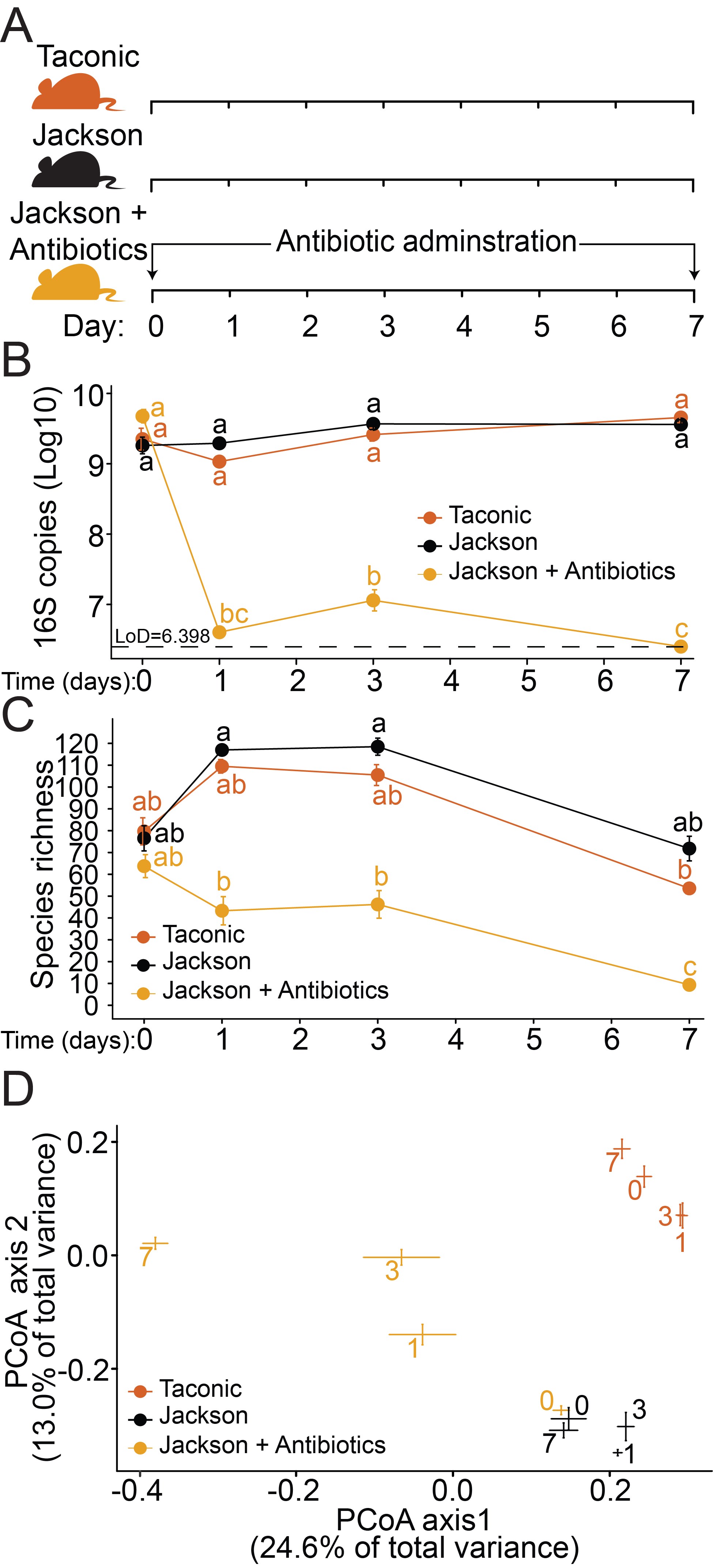

Supplement: Figure_S1_2025_11_06_wraf256 [file figure_s1_2025_11_06_wraf256.jpeg]

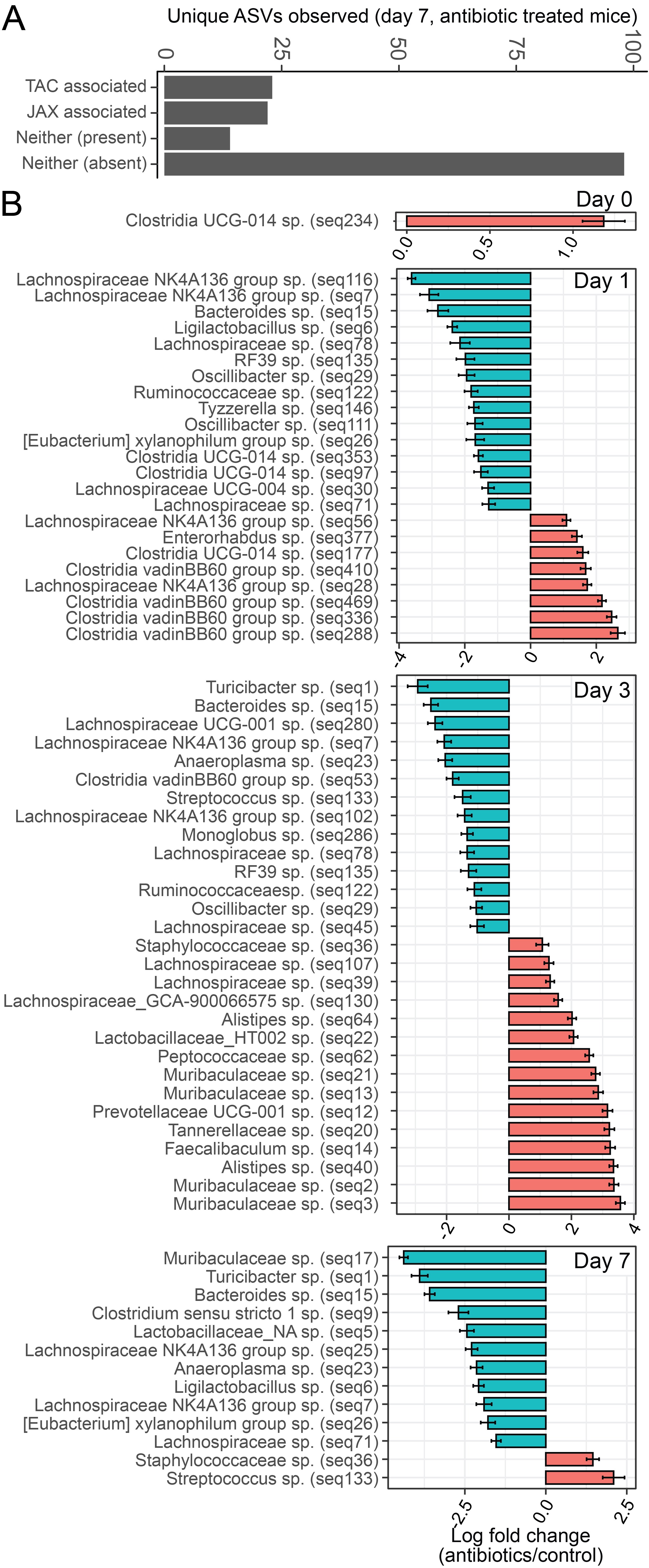

Supplement: Figure_S2_2025_11_06_wraf256 [file figure_s2_2025_11_06_wraf256.jpeg]

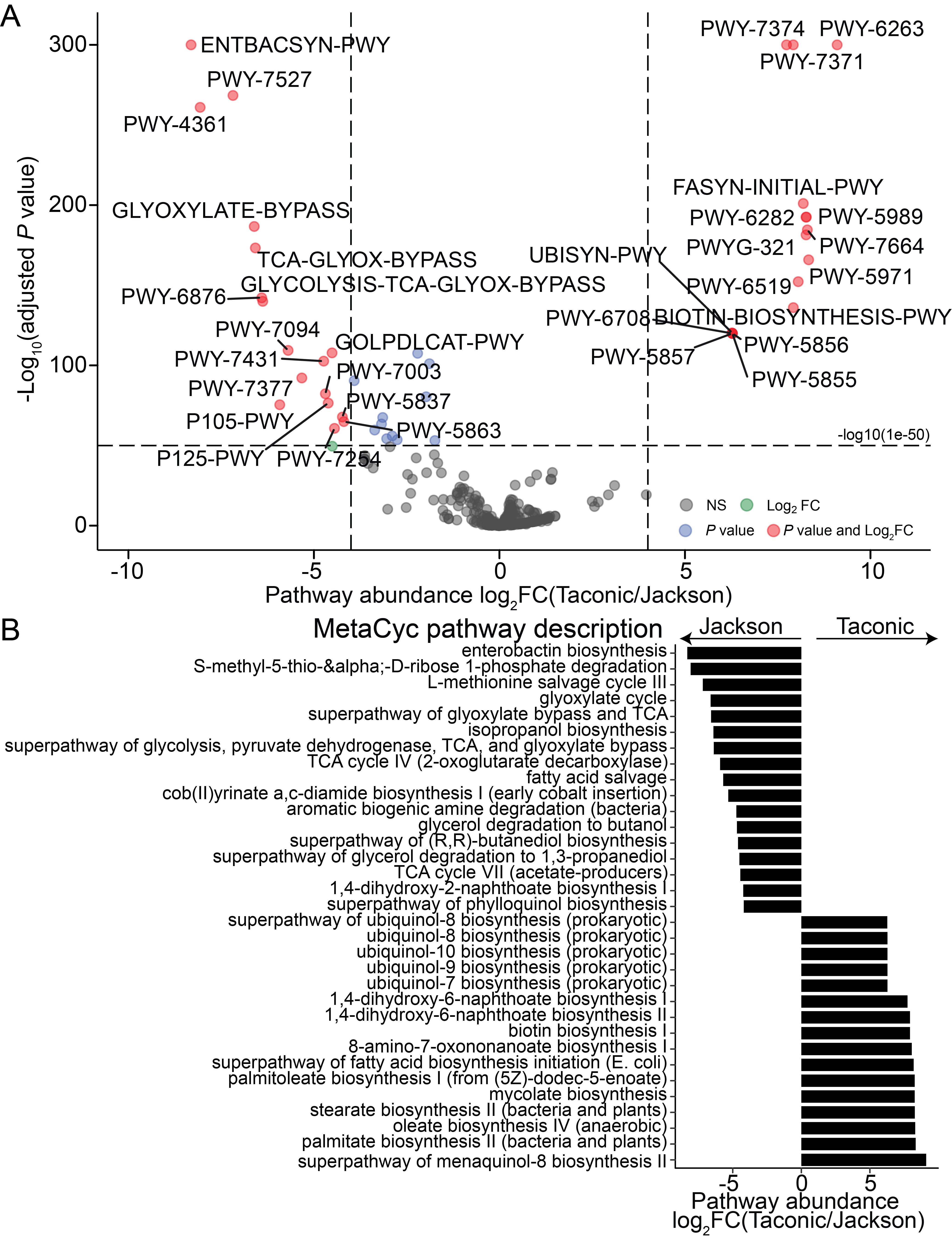

Supplement: Figure_S3_2025_11_06_wraf256 [file figure_s3_2025_11_06_wraf256.jpeg]

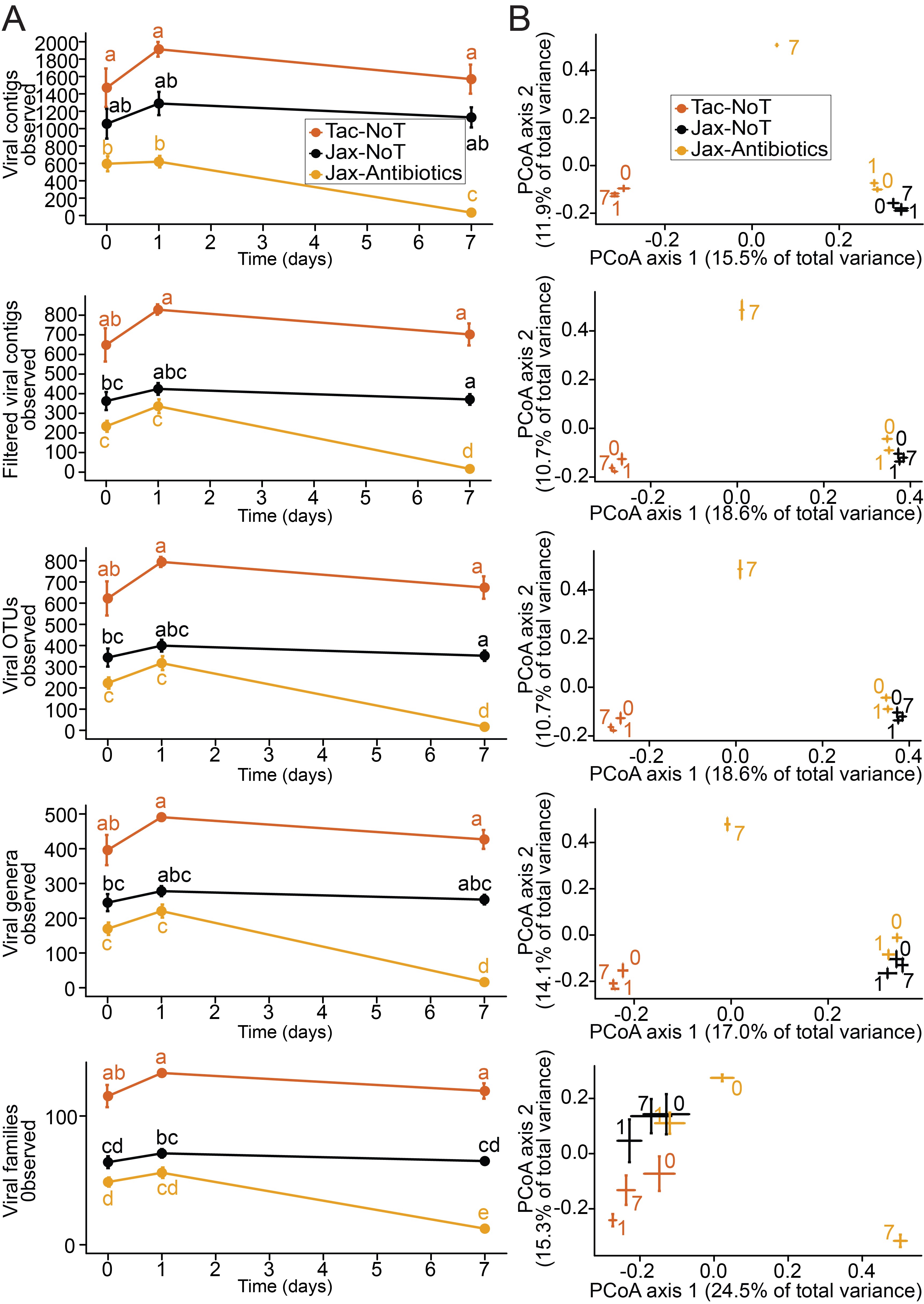

Supplement: Figure_S4_2025_11_06_wraf256 [file figure_s4_2025_11_06_wraf256.jpeg]
