# Supplementary material for: Differential microbial community assembly following co-housing versus microbiota transplant
Source: ISME J. 2025 Nov 17;19(1):wraf256. doi: 10.1093/ismejo/wraf256 (PMC12694414; doi:10.1093/ismejo/wraf256)
Supplement: Supplementary_Figure_Legends_wraf256 [file supplementary_figure_legends_wraf256.docx]

**Supplementary Figure 1.** **One-week treatment with antibiotic cocktail significantly alters faecal bacterial biomass and community composition.** (**A**) Mice were obtained from Jackson or Taconic labs. Mice from Jackson Labs were provided vancomycin, neomycin, and ampicillin in their drinking water for one week (Jackson + Antibiotics; n=13, 16, and 16 in three independent experiments). Untreated Jackson (n=~4 per experiment each) control mice and Taconic (n=~4 per experiment) mice were maintained on normal drinking water. (**B**) Bacterial load measured by qPCR of the 16S rRNA gene throughout the first week of the experiment. The letters next to each point indicate significant groupings, as those with the same letter were not significantly different from one another by two-way ANOVA followed by Tukey’s HSD post-hoc test for pairwise comparisons with an alpha of 0.05. (**C**) Bacterial species richness, the number of unique bacteria species observed per mouse, throughout the first week of the experiment via V4-16S rRNA gene sequencing. The letters next to each point indicate significant groupings, as those with the same letter were not significantly different from one another by Kruskal-Wallis followed by Bonferroni’s correction for multiple comparisons with an alpha threshold of 0.05. (**D**) Principal Coordinate Analysis of unweighted UniFrac distances calculated from species counts. Error bars (± SE) intersect at centroid of all samples collected for each group of mice on the day post-antibiotics treatment, indicated by the number on the plot.

**Alt text:** Plots display the impact of a one-week antibiotic treatment on bacterial load, diversity, and community structure. Antibiotic-treated mice show significant reductions in bacterial biomass and altered community composition compared to untreated controls.

**Supplementary Figure 2. Persistent ASVs in antibiotic-treated mice.** (**A**) Bar plot showing the number of ASVs detected on day 7 in antibiotic-treated mice, grouped by source classification. ASVs were defined as TAC-associated or JAX-associated based on prevalence and abundance in untreated controls collected during the first week of the experiment. ASVs not significantly associated with either vendor were subclassified as “Neither (present)” if observed in untreated controls but not significantly associated with either group, or “Neither (absent)” if absent from all control mice during the first week.

(**B**) Horizontal bar plots showing log fold change in bacterial species abundance between antibiotic-treated and untreated JAX mice across days 0, 1, 3, and 7, as determined by ANCOM-BC2. Bar length indicates the magnitude of log fold change, with positive values representing enrichment in antibiotic-treated mice and negative values representing enrichment in untreated controls. Error bars denote standard error. Only significantly enriched species with an absolute log fold change > 1 are plotted.

**Alt text**: Panel A is a horizontal bar plot where bar length represents the number of ASVs detected on day 7 in antibiotic-treated mice, grouped into four categories: JAX-associated, TAC-associated, “Neither (present),” and “Neither (absent).” Neither (absent) ASVs were the most prevalent in antibiotic treated mice on day 7. Panel B consists of four horizontal bar plots (days 0, 1, 3, and 7). Each bar represents a bacterial species, with bar length indicating log fold change in abundance between antibiotic-treated and untreated mice. Only one species was significantly enriched on day 0, primarily Clostridial species were enriched in antibiotic treated mice on day 1, Bacteroidetes on Day 3, and only one Staphylococcaceae and one Streptococcus species were enriched in antibiotic treated mice on day 7.

**Supplementary Figure 3. Predicted functional differences between JAX and TAC control mice.** (**A**) Volcano plot showing PICRUSt2-predicted functional capacities of fecal bacterial communities from JAX and TAC mice over the first week of the experiment. The x-axis shows log₂ fold change in predicted pathway abundance (Taconic vs. Jackson), and the y-axis shows -log_10_ adjusted *P* value. Each point represents a MetaCyc pathway. Colors indicate whether pathways were significant for fold change (green), *P* value (blue), both (red), or not significant (grey). (**B**) Bar plot showing selected MetaCyc pathway descriptions with their relative enrichment across vendors. The x-axis shows log₂ fold change in predicted pathway abundance (Taconic vs. Jackson), and bar length indicates the magnitude of fold change. Only pathways with an absolute log₂ fold change >3 and -log_10_ adjusted *P* value >50 are shown.

**Alt text**: Panel A is a volcano plot of predicted MetaCyc pathways comparing JAX and TAC control mice over the first week of the experiment. Panel B shows a bar chart of these pathways, with leftward bars indicating higher abundance in Jackson and rightward bars indicating higher abundance in Taconic. Jackson mice are enriched for pathways including enterobactin biosynthesis, methionine salvage, and the glyoxylate bypass, whereas Taconic mice are enriched for fatty acid and ubiquinol biosynthesis pathways

**Supplementary Figure 4.** **One-week treatment with antibiotic cocktail significantly alters faecal viral richness and community composition.** (**A**) Viral richness and (**B**) Principal Coordinate Analysis of Jaccard distances calculated from contig read counts of unique viral contigs observed per mouse, throughout the first week of the experiment via shotgun metagenomics sequencing of viral-like particles from feces. Panels represent data before i) and after contigs had been stringently filtered and grouped into clusters representing ii) strains, iii) species, iv) genera, and v) families. In A, the dot indicates the mean richness for that group, error bars (± SE), in B, the error bars (± SE) intersect at the centroid of all samples collected for each group of mice on the day indicated by the number on the plot. In A, the letters next to each point indicate significant groupings, those with the same letter were not significantly different from one another by pairwise Kruskal-Wallis followed by Bonferroni’s correction for multiple comparisons with an alpha threshold of 0.05. n = 3-8 samples per treatment group and timepoint.

**Alt text**: Viral richness and community composition are altered by antibiotics. Richness plots and PCoA based on Jaccard distances show reduced viral diversity post-treatment across multiple taxonomic levels. Viral contigs were clustered into different taxonomic levels as described in the Material and Methods section.
